# Strategies to inTerrupt RAbies Transmission for the Elimination Goal by 2030 In China (STRATEGIC): a modelling study

**DOI:** 10.1186/s12916-023-02821-x

**Published:** 2023-03-16

**Authors:** Qiulan Chen, Qiuping Liu, Chao Gong, Wenwu Yin, Di Mu, Yu Li, Shujun Ding, Yifang Liu, Hao Yang, Shuwu Zhou, Sa Chen, Zhongfa Tao, Yanping Zhang, Xun Tang

**Affiliations:** 1 Key Laboratory of Surveillance and Early-Warning On Infectious Disease, Chinese Center for Disease Control and Prevention, Beijing, 102206 China; 2 Department of Epidemiology and Biostatistics, Peking University School of Public Health, Beijing, 100191 China; 3 Shandong Center for Disease Control and Prevention, Jinan, China; 4 Tianjin Centers for Disease Control and Prevention, Tianjin, China; 5 Hunan Provincial Center for Disease Control and Prevention, Changsha, China; 6 Guangxi Zhuang Autonomous Region Center for Disease Prevention and Control, Nanning, China; 7 Shaanxi Provincial Center for Disease Control and Prevention, Xi’an, China; 8 Guizhou Center for Disease Control and Prevention, Guiyang, China

**Keywords:** Cost-effectiveness analysis, Dog-mediated human rabies, Neglected tropical diseases, Decision-analytic modelling, Chinese

## Abstract

**Background:**

A global plan has been set to end human deaths from dog-mediated rabies by 2030 ("Zero-by-30"), but whether it could be achieved in some countries, such as China, remains unclear. Although elimination strategies through post-exposure prophylaxis (PEP) use, dog vaccination, and patient risk assessments with integrated bite case management (IBCM) were proposed to be cost-effective, evidence is still lacking in China. We aim to evaluate the future burdens of dog-mediated human rabies deaths in the next decade and provide quantitative evidence on the cost-effectiveness of different rabies-control strategies in China.

**Methods:**

Based on data from China's national human rabies surveillance system, we used decision-analytic modelling to estimate dog-mediated human rabies death trends in China till 2035. We simulated and compared the expected consequences and costs of different combination strategies of the status quo, improved access to PEP, mass dog vaccination, and use of IBCM.

**Results:**

The predicted human rabies deaths in 2030 in China will be 308 (95%UI: 214–411) and remain stable in the next decade under the status quo. The strategy of improved PEP access alone could only decrease deaths to 212 (95%UI: 147–284) in 2028, remaining unchanged till 2035. In contrast, scaling up dog vaccination to coverage of 70% could eliminate rabies deaths by 2033 and prevent approximately 3,265 (95%UI: 2,477–3,687) extra deaths compared to the status quo during 2024–2035. Moreover, with the addition of IBCM, the "One Health" approach through mass dog vaccination could avoid unnecessary PEP use and substantially reduce total cost from 12.53 (95%UI: 11.71–13.34) to 8.73 (95%UI: 8.09–9.85) billion US dollars. Even if increasing the total costs of IBCM from 100 thousand to 652.10 million US dollars during 2024–2035, the combined strategy of mass dog vaccination and use of IBCM will still dominate, suggesting the robustness of our results.

**Conclusions:**

The combined strategy of mass dog vaccination and IBCM requires collaboration between health and livestock/veterinary sectors, and it could eliminate Chinese rabies deaths as early as 2033, with more deaths averted and less cost, indicating that adding IBCM could reduce unnecessary use of PEP and make the "One Health" rabies-control strategy most cost-effective.

**Supplementary Information:**

The online version contains supplementary material available at 10.1186/s12916-023-02821-x.

## Background

As a nearly always fatal but entirely vaccine-preventable viral disease, dog-transmitted human rabies remains a critical but neglected threat to global health in some countries, especially for children [1]. For example, given the vast population size, besides India, China reported the second-largest number of global rabies deaths [2], and rabies is still the second most common infectious cause of death for Chinese children, despite an annual decrease of 20% over the past decade [3]. In many countries, rabies elimination was successfully achieved through vaccination for both human and domestic dogs by the "One Health" approach [4]. In this regard, the World Health Organization (WHO) and partners have set a goal that, by 2030, up to 92% of countries in the world could eliminate dog-mediated human rabies deaths ("Zero-by-30"), and all nations would reduce dog-mediated human rabies deaths by 50% [5]. However, whether the global goal of "Zero-by-30" could be achieved in some countries, such as China, is still undetermined.

The WHO rabies modelling consortium has developed epidemiological and economic models to predict rabies deaths and investigate the effectiveness of enhancing post-exposure prophylaxis (PEP) access in 67 rabies-endemic countries during 2020–2035 [6]. This modelling study supported the "One Health" approach and suggested that mass dog vaccination programmes could eliminate dog-mediated rabies over this period, and improved PEP access was highly cost-effective, especially in combination with patient risk assessments by the integrated bite case management (IBCM). Unfortunately, as a populous and rabies-endemic country, China is underrepresented in this global modelling study.

Unlike other Western or Gavi-eligible nations [7], China has unique but complex health systems, with multiple levels of care and various health providers offering different services [8]. For example, PEP treatment for patients with potential rabies exposures is available at county-level centers for disease control and prevention, hospitals and township-based rabies clinics (health sector). China's current availability of PEP use seems sufficient, while patient risk assessments by the IBCM are not yet implemented [9]. In some provinces with high rabies burdens, such as Guizhou, Hunan and Guangxi, public insurance of the New Rural Cooperative Medical Scheme (NRCMS) covered PEP-related medical expenses and has contributed to the observed decline of human rabies cases in rural areas. However, most provinces in China have discontinued the reimbursement of PEP following the integration of the NRCMS and the Urban Resident Basic Medical Insurance in 2016. As a result, patients currently pay the whole PEP series as an out-of-pocket cost, which might hinder their possibility of adherence to the PEP treatment regimen [10].

On the other hand, dog vaccination coverage in China remains inadequate from the "One Health" approach perspective [10, 11]. As a nationally notifiable disease for both dogs and humans, dog-mediated rabies management involves multiple sectors in China [9], including the health sector responsible for human PEP and immunization programmes, the livestock/veterinary sector responsible for supplying veterinary rabies vaccine and implementing mass dog vaccination, and public security departments responsible for pet dog registration, vaccination, and stray dog management in cities. It is estimated that there are approximately 80–100 million dogs in China [9]. Before the revision and implementation of the Animal Epidemic Prevention Law of the People's Republic of China in 2021, there were no national, unified regulations for dog management and dog vaccination against rabies in China. As a result, dog management practices were not optimal, and the registration and vaccination rate was low, particularly in rural areas [12].

Taken togehter, evidence on the effectiveness and cost-effectiveness of comprehensive strategies of improved PEP access for humans, mass dog vaccination, and collaboration between the health and livestock/veterinary sectors through the use of IBCM in the context of the "One Health" approach is lacking in China. We used the updated data from China's national human rabies surveillance system and similar decision-analytic modelling methods as the WHO rabies modelling consortium in the Strategies to inTerrupt RAbies Transmission for the Elimination Goal by 2030 In China (STRATEGIC) study. Specific aims include: (1) to estimate whether the global rabies plan of "Zero-by-30" could be achieved in China under the status quo; (2) if not, to predict the time when the Chinese elimination goal will be accomplished; and (3) to find out the most cost-effective strategy for the purpose in China.

## Methods

As described in the statistical analysis plan (Additional file 1: Statistical Analysis Plan) [6, 13–15], we adopted a decision tree model to simulate human rabies dynamics in different strategies for rabies control, similar to the WHO rabies modelling consortium study for direct comparisons [6]. We used data collected by the Chinese Center for Disease Control and Prevention (China CDC) in the National Human Rabies Surveillance (NHRS) system and also in some provincial surveillance points, including Shandong (East China), Hunan (Central China), Tianjin (North China), Guangxi (South China), Shaanxi (Northwest China) and Guizhou (Southwest China), to investigate potential regional disparities in diverse areas (Additional file 1: Fig. S1). Our study followed the updated Consolidated Health Economic Evaluation Reporting Standards 2022 (CHEERS 2022) checklist [13] (Additional file 1: CHEERS 2022 Checklist). All analyses were performed in R (Version 4.0.5).

### Scenarios

**Fig. 1:**
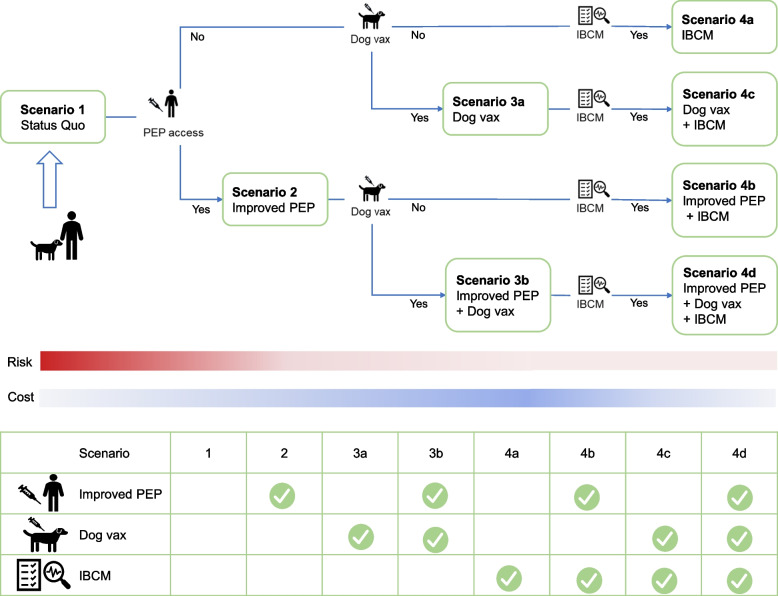
The conceptual diagram of the decision tree and scenarios. PEP, post-exposure prophylaxis; IBCM, integrated bite case management; Dog vax, mass dog vaccination

We considered four primary scenarios (Fig. 1 and Additional file 1: Table S1). (1) status quo: rabies prevention is performed according to the current practice in China without IBCM as usual, i.e., victims bitten by dogs and seek PEP treatment in clinics and paid by themselves, while mass dog vaccination remains low, below 70%; (2) expanding PEP access: we assumed that basic health insurance would cover the cost of PEP treatment to increase the probability of health-seeking, receiving and completing PEP treatment; (3) scaling up mass dog vaccination coverage: we assumed that the number of rabid dogs would decrease as dog vaccination coverage increased to 70%, (especially in rural areas by livestock/veterinary sector), as recommended by the WHO [2]. Two sub-scenarios were included: (3a) increased mass dog vaccination coverage based on the status quo, and (3b) increased mass dog vaccination coverage in addition to expanding PEP access. Last but not least, (4) Use of the IBCM: we evaluated the impact of the IBCM approach, where the health sector and livestock/veterinary sector collaborate for the risk assessment of patients bitten by dogs. Four sub-scenarios were considered: (4a) IBCM with current PEP provision according to the status quo, (4b) IBCM with improved free PEP access only, (4c) IBCM with mass dog vaccination only, and (4d) IBCM with enhanced free PEP and mass dog vaccination. All strategies were assumed to start from 2024.

### Decision tree model

The decision tree model (Additional file 1: Fig. S2) was used to obtain health outcomes and direct medical costs by simulating the behaviour of a person seeking medical care after being bitten by a dog, with parameters from published literature, expert consultation, and data from the national human rabies surveillance system. In the tree model, the person might be bitten by a rabid or healthy dog and then decide whether to seek medical care for receiving and completing the PEP treatment. Only those bitten by the rabid dog will die from rabies, and the probability of dying from rabies could be reduced by PEP treatment.

The cost-effectiveness analysis was done from the perspective of the policymaker. We measured health outcomes by human rabies deaths, disability-adjusted life-years (DALYs), and calculated costs by direct medical expenses only. The incremental cost-effectiveness ratio (ICER) was reported in terms of cost per death prevented, converting all prices to US dollars in 2020. Based on China's per-capita gross domestic product (GDP) of 10,410 US dollars in 2020, if the ICER is less than three times per-capita GDP, the strategy is considered "cost-effective". All scenarios were simulated with a discount rate of 3%, and the time horizon was set from 2024 to 2035.

### Model parameters and assumptions

The parameters were divided into three groups based on their role in being bitten by dogs, seeking medical care, and the resulting health outcomes for patients. These categories include parameters related to rabies exposure, healthcare activities, and DALYs and costs. Parameters related to rabies exposure were used to determine the number of people bitten by rabid or healthy dogs. Those related to health care activities were used to calculate the number of human deaths due to dog-borne rabies and medical services used. The parameters related to DALYs and costs were used to calculate the DALYs and estimate the unit cost (i.e., the price weight). The parameter values (including the probability distributions of each probability function) are presented in Additional file 1: Table S2-S4 [6, 9, 16–28].

#### Parameters related to the rabies exposure

The probabilities and numbers bitten by dogs were calculated as follows:

1 $${P}_{bitten\;by\;rabid\;dog}=\frac{{n}_{dog\;in\;2020}\times {P}_{rabid|dog}\times {P}_{bite|rabid\;dog}}{{n}_{man\;in\;2020}}$$

2 $${P}_{bitten\;by\;healthy\;dog}=\frac{{n}_{bitten\;by\;all\;dog\;in\;2020}-{n}_{dog\;in\;2020}\times {P}_{rabid|dog}\times {P}_{bite|rabid\;dog}}{{n}_{man\;in\;2020}}$$

3 $${n}_{bitten\;by\;rabid\;dog}={n}_{man}\times {P}_{bitten\;by\;rabid\;dog}$$

4 $${n}_{bitten\;by\;healthy\;dog}={n}_{man}\times {P}_{bitten\;by\;healthy\;dog}$$

In the above formulas, $${n}_{man}$$ represents the number of human population. The number in 2020 was obtained from the National Bureau of Statistics of China [16]. The number of dog population, represented by $${n}_{dog}$$, was estimated based on the human population and a constant human-to-dog ratio of 14. It was assumed that the ratio of humans to dogs remained constant over time. The human-to-dog ratio was calculated by dividing the total human population in 2020 (1,412 million) by the number of dogs in the same year (100 million) [9]. We assumed a stable human birth rate (0.852%) and mortality rate (0.707%) to simulate the number of human population from 2021 to 2035. The parameter $${n}_{bitten by all dog}$$ (7.78 million) represents the number of human population bitten by all dogs, was obtained from first-visit cases in rabies PEP clinics from the NHRS system [9]. The rabies incidence in dogs ($${P}_{rabid|dog}$$=0.0003) was estimated by expert consultation based on data from the first Chinese Rabies Surveillance Plan in animal populations during 2004–2018 [18]. Because the average number of bites per rabid dog ($${P}_{bite|rabid dog}$$=0.38) is currently unavailable in China, we used the same value as the WHO Rabies Modelling Consortium study [6], for international comparison.

The parameter $${P}_{bitten by rabid dog}$$ represents the probability of a patient being bitten by a rabid dog. It was calculated using Formula (1) by dividing the total human population in 2020 by the number of people bitten by rabid dogs that year. The number of rabid dogs in 2020 was obtained by multiplying the dog population by the rabid incidence in dogs. The number of people bitten by rabid dogs was estimated by multiplying the number of rabid dogs by the average number of bites per rabid dog. We assumed that the probability of being bitten by a rabid dog would remain constant over time and was used to estimate the number of people bitten by rabid dogs beyond 2020 (Formula 3).

The probability of a patient being bitten by healthy dogs is represented by the parameter $${P}_{bitten by healthy dog}$$. We assumed that this probability would remain constant over time and was used to estimate the number of patients bitten by healthy dogs beyond the year 2020 (Formula 4). This value was calculated by dividing the human population in 2020 by the number of patients bitten by healthy dogs that year, as per Formula (2). The number of patients bitten by healthy dogs in 2020 was determined by subtracting the number of patients bitten by rabid dogs from the total number of patients bitten by all dogs ($${n}_{bitten by all dog}$$).

#### Parameters related to the health care activities

Human deaths caused by dog-mediated rabies were calculated as follows:

5 $${n}_{deaths}={P}_{infect}\times ({n}_{bitten\;by\;rabid\;dog}-{n}_{bitten\;by\;rabid\;dog}\times {P}_{seek}\times {P}_{receive1}\times {P}_{complete}\times \left({P}_{prevent|complete}\times \left(1-{P}_{receive2}\right)+{P}_{prevent|rig}\times {P}_{receive2}\right)-{n}_{bitten\;by\;rabid\;dog}\times {P}_{seek}\times {P}_{receive1}\times \left(1-{P}_{complete}\right)\times {P}_{prevent|incomplete})$$

We used the probabilities of seeking medical care (${P}_{seek}$=0.85), receiving PEP treatment (${P}_{receive1}$=0.99), receiving rabies immunoglobulin (RIG) (${P}_{receive2}$=0.17), and completing the PEP regimen (${P}_{complete}$=0.91) from the NHRS system 21, to describe the behaviours of health care activities for patients bitten by a dog. We assumed that ${P}_{seek}$, ${P}_{receive1}$ and ${P}_{complete}$ would change with the improvement of PEP access by a 0.01 increment per year to a cap of 0.9, 0.99 and 0.975, respectively. Consistent with the WHO Rabies Modelling Consortium study [6], the ${P}_{receive1}$ would drop by 50% and 90% with IBCM before and after the rabies elimination, respectively. For patients bitten by rabid dogs, the following parameters were used to calculate the probabilities of dying from rabies: the probability of developing rabies without any intervention (${P}_{infect}$=0.16), the probability of avoiding rabies given a complete PEP (${P}_{prevent|complete}$=1), the probability of avoiding rabies given an incomplete PEP (${P}_{prevent|incomplete}$=0.99) [25], and the probability of avoiding rabies given an RIG injection (${P}_{prevent|rig}$=1).

#### Parameters related to DALYs and costs

Consistent with the WHO Rabies Modelling Consortium study [6], we estimated the mean DALY caused by rabies using data on the age distribution of the human rabies deaths and age-specific life expectancy. Age distribution of the human rabies deaths during 2011–2021 was taken from the NHRS system. The life expectancy in 2024 was estimated by a life table (Additional file 1: Table S5), obtained from the United Nations World Population Prospects 2022 [29]. According to the standard PEP treatment procedure, we only considered the direct costs: registration fee of the first visit, injection fee, costs of wound cleaning, human rabies vaccines, RIG, and dog vaccines (Additional file 1: Table S2). All costs were converted to US dollars at the exchange rate in 2020 (6.8996 Chinese Yuan per 1 US dollar), with a discount rate of 3%.

### Sensitivity analysis

We performed probabilistic sensitivity analyses (PSA) to examine the robustness of our results. By drawing 1,000 sets of model parameter values from their distributions, we constructed the results' 95% uncertainty interval (UI). We also separately considered the uncertainty of the following parameters: (1) incidence of rabid dog bites per person annually (the rabid bite incidence); (2) incidence of non-rabid dog bites per person annually (the non-rabid bite incidence); (3) probability of developing rabies with exposure (*P*_infect_); and (4) probability of preventing rabies by complete or incomplete PEP treatment (*P*_prevent_) in the one-way sensitivity analyses.

## Results

**Fig. 2:**
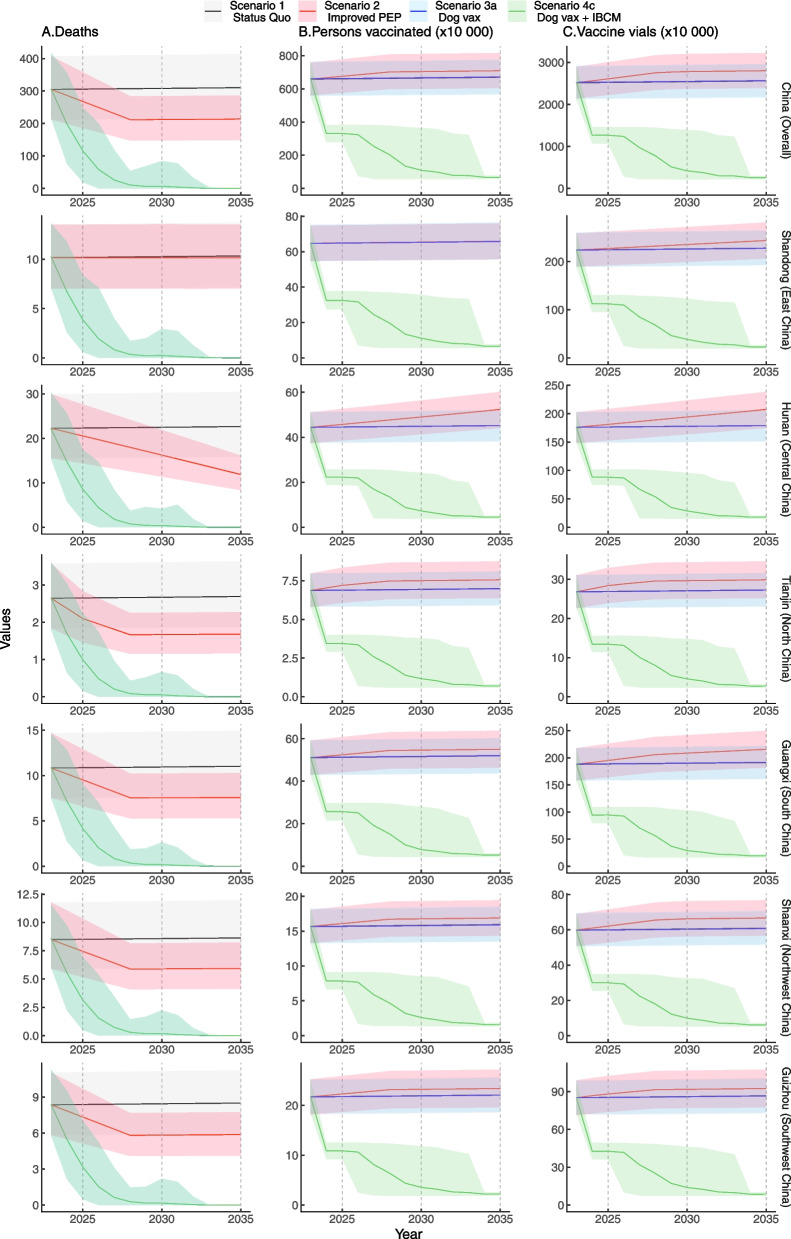
Trends of the predicted human rabies deaths and vaccine vials during 2023–2035 in China. Under the status quo, the predicted number of dog-mediated human rabies deaths will remain constant in 2030. However, scaling up dog vaccination could facilitate the elimination goal in national and area-specific analyses, suggesting that human rabies deaths will be ended as early as 2026 in Tianjin (North China). Scenario 1, Status quo; Scenario 2, Improved PEP; Scenario 3a, Improved mass dog vaccination; Scenario 3b, Improved PEP + improved mass dog vaccination; Scenario 4a, Status quo + IBCM; Scenario 4b, Improved PEP + IBCM; Scenario 4c, Improved mass dog vaccination + IBCM; Scenario 4d, Improved PEP + improved mass dog vaccination + IBCM. PEP, post-exposure prophylaxis; IBCM, integrated bite case management

Cumulatively 8308 dog-mediated human rabies deaths in China were reported during 2011–2020, with declining trends annually (Additional file 1: Fig. S3). However, vast regional rabies-epidemic disparities were shown: most deaths (1071) were in Guangxi (South China), while the rabies cases in Tianjin (North China) were much fewer (36) (Additional file 1: Fig. S1). Totally 146.21 million vials of human-used PEP vaccines were signed in the same period of 2011–2020, and the annual amount in 2020 was 17.55 million, suggesting adequate access to PEP in China by far. Maintaining the current level of PEP access in China is expected to avert 19,270 (95%UI: 13,459–25,921) deaths or 304,106 (95%UI: 212,402–409,064) DALYs from 2024 to 2035, compared to the condition without PEP (Table 1). However, under the status quo, the predicted number of dog-mediated human rabies deaths will remain stable at 308 (95%UI: 214–411) in 2030 (Table 2 and Fig. 2), with a total of 3,695 (95%UI: 2,572–4,931) deaths or 58,317 (95%UI: 40,581–77,817) DALYs in China in the next decade.

**Table 1:** Trends of dog-mediated human rabies burden during 2024–2035 under different scenarios in China

**Scenario**	**Rabies deaths**	**Rabies deaths averted**	**DALYs**	**DALYs averted**	**Human vaccine vials used** **(in millions)**	**Total cost** **(US dollars, in billions)**
Scenario 1	3,695 (2,572–4,931)	19,270 (13,459–25,921)	58,317 (40,581–77,817)	304,106 (212,402–409,064)	304.5658 (259.7306–350.5717)	9.3839 (8.6159–10.1720)
Scenario 2	2,737 (1,904–3,663)	20,229 (14,132–27,209)	43,616 (30,336–58,367)	318,806 (222,719–428,815)	327.9403 (279.6642–377.4770)	9.7075 (8.8919–10.5445)
Scenario 3a	429 (95–1,244)	2,236 (495–6,487)	7,691 (1,754–21,532)	40,121 (9,115–112,314)	305.5471 (257.7704–352.9162)	12.5258 (11.7074–13.3373)
Scenario 3b	376 (88–1,025)	2,289 (502–6,707)	6,762 (1,625–17,879)	41,050 (9,244–115,973)	328.9948 (277.5512–379.9990)	12.8505 (11.9813–13.7123)
Scenario 4a	3,695 (2,572–4,931)	19,270 (13,459–25,921)	58,317 (40,581–77,817)	304,106 (212,402–409,064)	152.5057 (130.0681–175.5384)	6.7791 (6.3947–7.1736)
Scenario 4b	2,737 (1,904–3,663)	20,229 (14,132–27,209)	43,616 (30,336–58,367)	318,806 (222,719–428,815)	164.2100 (140.0504–189.0104)	6.9411 (6.5329–7.3602)
Scenario 4c	429 (95–1,244)	2,236 (495–6,487)	7,691 (1,754–21,532)	40,121 (9,115–112,314)	79.2837 (43.6736–145.5069)	8.7319 (8.0869–9.8504)
Scenario 4d	376 (88–1,025)	2,289 (502–6,707)	6,762 (1,625–17,879)	41,050 (9,244–115,973)	84.1877 (46.1345–156.1162)	8.804 (8.1223–10.0017)

Scenario 1, Status quo; Scenario 2, Improved PEP; Scenario 3a, Improved mass dog vaccination; Scenario 3b, Improved PEP + improved mass dog vaccination; Scenario 4a, Status quo + IBCM; Scenario 4b, Improved PEP + IBCM; Scenario 4c, Improved mass dog vaccination + IBCM; Scenario 4d, Improved PEP + improved mass dog vaccination + IBCM. PEP, post-exposure prophylaxis; IBCM, integrated bite case management

**Table 2:** The predicted number of dog-mediated human rabies deaths during 2023–2035 in China by regions

Scenario	Scenario 1	Scenario 2	Scenario 3a	Scenario 3b
**China (Overall)**				
Year 2023	305 (212–407)	305 (212–407)	306 (217–417)	306 (217–417)
Year 2025	306 (213–408)	268 (186–358)	116 (19–249)	102 (17–218)
Year 2028	307 (214–410)	212 (147–284)	10 (0–54)	7 (0–37)
Year 2030	308 (214–411)	212 (148–284)	7 (0–84)	5 (0–58)
Year 2033	310 (215–413)	213 (148–286)	0 (0–3)	0 (0–2)
**Shandong (East China)**				
Year 2023	10 (7–14)	10 (7–14)	10 (7–14)	10 (7–14)
Year 2025	10 (7–14)	10 (7–14)	4 (1–8)	4 (1–8)
Year 2028	10 (7–14)	10 (7–14)	0 (0–2)	0 (0–2)
**Hunan (Central China)**				
Year 2023	22 (16–30)	22 (16–30)	22 (15–30)	22 (15–30)
Year 2025	22 (16–30)	21 (14–28)	9 (1–17)	8 (1–16)
Year 2029	22 (16–30)	17 (12–23)	0 (0–5)	0 (0–3)
**Tianjin (North China)**				
Year 2023	3 (2–4)	3 (2–4)	3 (2–4)	3 (2–4)
Year 2025	3 (2–4)	2 (1–3)	1 (0–2)	1 (0–2)
Year 2026	3 (2–4)	2 (1–3)	0 (0–2)	0 (0–1)
**Guangxi (South China)**				
Year 2023	11 (8–15)	11 (8–15)	11 (8–15)	11 (8–15)
Year 2025	11 (8–15)	10 (7–13)	4 (1–9)	4 (1–8)
Year 2028	11 (8–15)	8 (5–10)	0 (0–2)	0 (0–1)
**Shaanxi (Northwest China)**				
Year 2023	8 (6–12)	8 (6–12)	9 (6–12)	9 (6–12)
Year 2025	9 (6–12)	7 (5–10)	3 (0–7)	3 (0–6)
Year 2028	9 (6–12)	6 (4–8)	0 (0–1)	0 (0–1)
**Guizhou (Southwest China)**				
Year 2023	8 (6–11)	8 (6–11)	8 (6–11)	8 (6–11)
Year 2025	8 (6–11)	7 (5–10)	3 (0–7)	3 (0–6)
Year 2027	8 (6–11)	6 (4–8)	1 (0–3)	0 (0–2)

Scenario 1, Status quo; Scenario 2, Improved PEP; Scenario 3a, Improved mass dog vaccination; Scenario 3b, Improved PEP + Improved mass dog vaccination. PEP, post-exposure prophylaxis; IBCM, integrated bite case management

If expanding PEP access in China (scenario 2), the annual deaths will decrease from 305 (95%UI: 212–407) in 2023 to 212 (95%UI: 147–284) in 2028 and then remain unchanged till 2035 (Table 2). Total deaths and DALYs will reduce to 2,737 (95%UI: 1,904–3,663) and 43,616 (95%UI: 30,336–58,367), respectively. Compared to the status quo, this strategy of improving PEP access alone could prevent approximately 958 (95%UI: 668–1,268) extra deaths during 2024–2035 (Table 1). In contrast, scaling up dog vaccination to the coverage of 70% could eliminate rabies deaths as soon as 2033 in China. With mass dog vaccination alone (scenario 3a), the cumulative number of deaths will reduce to 429 (95%UI: 95–1,244), thus averting approximately 3,265 (95%UI: 2,477–3,687) extra deaths in contrast to the status quo during 2024–2035. Under scenario 3a, by 2025, Chinese human rabies deaths will decrease to 116 (95%UI: 19–249), which is half of the counterpart in 2023 (i.e., achieving the global goal of a 50% reduction in human rabies mortality). Of note, even by combining improved PEP access and mass dog vaccination (scenario 3b), the earliest time to eliminate the Chinese rabies deaths will remain in 2033.

Concerning costs, a total of 304.57 (95%UI: 259.73–350.57) million human-used PEP vaccines will be required during 2024–2035 under the status quo, approximately 25.38 million vials annually, which already exceeds the signed vials in 2020 in China (Table 1). Furthermore, an additional 23.37 (95%UI: 19.93–26.91) million vaccine vials with extra costs of 323.60 (95%UI: 276.00–372.50) million US dollars will be needed to expand PEP access (scenario 2) from 2024 to 2035. The trend of rabies deaths under the scenarios that included IBCM (scenarios 4a to 4d) was similar to those without IBCM (scenarios 1, 2, 3a and 3b) (Additional file 1: Table S6 and Fig. S4). However, the use of IBCM could substantially reduce the PEP vaccine under the status quo from 304.57 (95%UI: 259.73–350.57) to 152.51 (95%UI: 130.07–175.54) million vials during 2024–2035, with the total cost decreasing from 9.38 (95%UI: 8.62–10.17) to 6.78 (95%UI: 6.39–7.17) billion US dollars.

From the cost-effectiveness perspective, the ICERs of the strategies of improving PEP access only (scenario 2) or mass dog vaccination alone (scenario 3a) are 337,771 or 962,920 US dollars per death prevented, respectively, compared with the status quo (Additional file 1: Table S7). Although the ICERs of both strategy 2 and 3a exceed the cost-effectiveness threshold of three times China's GDP per capita, the strategy of IBCM and mass dog vaccination (scenario 4c) dominates the status quo, with more deaths averted and less cost. In addition, compared with scenario 4c, the ICER of the combination strategy of enhancing PEP access, mass dog vaccination, and IBCM (scenario 4d) is also beyond the cost-effectiveness threshold. Thus, the former strategy of combining mass dog vaccination and the use of IBCM (scenario 4c) is the most cost-effective among all the elimination strategies (i.e., scenarios 3a, 4c and 4d).

**Fig. 3:**
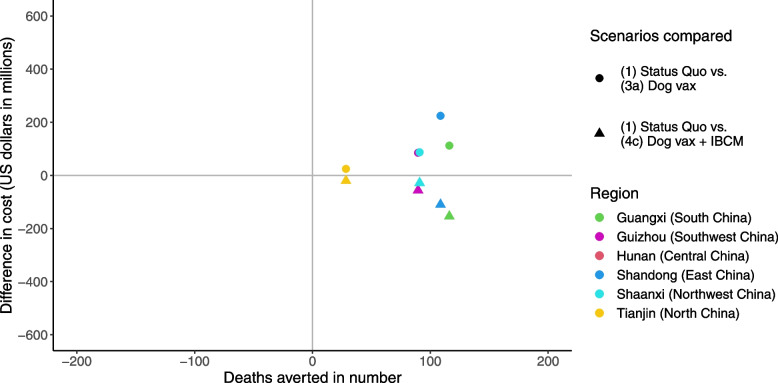
The cost-effectiveness plane for the incremental cost-effectiveness ratio per death prevented by areas in China. Area-specific cost-effectiveness analyses showed that the strategy combined with mass dog vaccination and IBCM (scenario 4c) could cost-effectively prevent rabid deaths, while the total cost was lower than the status quo. Scenario 1, Status quo; Scenario 3a, Improved mass dog vaccination; Scenario 4c, Improved mass dog vaccination + IBCM. PEP, post-exposure prophylaxis; IBCM, integrated bite case management

In terms of regional disparities for rabies control, under the status quo, the trends of dog-mediated human rabies deaths in different areas of China are broadly similar to those at the national level. However, with improved PEP access (scenario 2), the deaths in Hunan (Central China) continue to decline, while the trends in Shandong (East China) remain almost unchanged. Scaling up dog vaccination could also facilitate the elimination goal in our six region-stratified analyses, showing that human rabies deaths in all those areas will be zero by 2029 (Table 2 and Fig. 2), which would be earlier than the goal of "Zero-by-30". Moreover, in area-specific cost-effectiveness analyses (Fig. 3 and Additional file 1: Table S8-S13), the ICERs of strategies are generally consistent with those at the national level that the one combining mass dog vaccination and use of IBCM (scenario 4c) would be the most cost-effective.

Sensitivity analyses showed that estimates of the rabies burden of the strategy of mass dog vaccination (scenario 3a) and the one combining mass dog vaccination with the use of IBCM (scenario 4c) might be most affected by uncertainty in the incidence of rabid dog bites (Additional file 1: Fig. S5). However, strategies with mass dog vaccination (i.e., scenarios 3a, 3b, 4c, and 4d) coordinated the reduction of rabies burden, and it seems less likely to change the magnitude of estimates of dog-mediated human rabies deaths and DALYs, with shorter uncertainty intervals in PSA of 1000 times of simulations (Additional file 1: Fig. S6).

According to the WHO recommendation [28], the average cost of mass dog vaccination is estimated to be 4.03 US dollars per dog, ranging from 1.56 to 11.33 US dollars. Given the budget constraints, as the cost of mass dog vaccination per dog rises to 13.69, 13.81, and 14.06 US dollars, the additional cost (compared to the status quo) of the strategy combining both mass dog vaccination and IBCM will reach the level equivalent to zero, one, and three times China's per capita GDP, respectively. It highlights the possibility that if the cost of mass dog vaccination per dog can be kept below 14 US dollars, the strategy incorporating both mass dog vaccination and IBCM may become cost-effective in China. Even if increasing the total costs of IBCM from 100 thousand to 652.10 million US dollars during 2024–2035 (i.e., 8.33 thousand to 54.34 million US dollars annually), the combined strategy of mass dog vaccination and use of IBCM (scenario 4c) will still dominate. When the annual cost of IBCM exceeds 62.84 million US dollars (about 6036 times China's GDP per capita in the year 2020), the ICER of this strategy (scenario 4c) will exceed three times GDP per capita, suggesting the robustness of our conclusions.

## Discussion

Our study indicated that, under the status quo, the burden of human rabies in China would remain considerable in the next decade. The annual rabies deaths have decreased in recent years with much effort by the Chinese government, such as the launch of the National Animal Rabies Prevention and Control Plan (2017–2020) and the revision of the Animal Epidemic Prevention Law (2021) [9]. Nevertheless, the trends of the rabies epidemic and the purpose of elimination still call for specific, actionable strategies in China. Globally, the WHO and partners are also focusing on the key strategic framework to guide the cross-sectoral collaborative activities in the forthcoming Joint Plan of Action for One Health (2022–2026) [30]. On this matter, our STRATEGIC study may fill the gaps in evidence on comprehensive strategies for rabies control in China and promote the achievement of the global plan of "Zero-by-30".

The WHO Rabies Modelling Consortium study suggested expanding PEP access to be highly cost-effective in 67 rabies-endemic countries [6]; however, its effectiveness is quite limited in China for the elimination goal of "Zero-by-30". Improving PEP access alone could only reduce less than half of deaths but is not enough to eventually end the dog-mediated rabies epidemic in China. Unlike those Gavi-eligible countries, China's current supplies and coverage of PEP vaccines seemed adequate 9, for example, about 94.64% of the outpatients from rabies PEP clinics completed the whole schedule of doses in Tianjin [21]. In contrast, the national level of dog vaccination coverage is as low as 40% currently in China [10]. What is worse, dog vaccination coverage was just 19.1% in rural Guangxi (South China) in 2021 [31]. Thus, there is still room for improvement in dog vaccination coverage rather than merely expanding human PEP access in light of the "One Health" approach [32, 33]

The current study showed that it is expected to achieve the elimination goal in China as soon as 2033 by implementing mass dog vaccination coverage of 70%, highlighting dog vaccination in the "One Health" approach for rabies control, which is consistent with findings from other countries [34, 35]. In India, for instance, if scaling up dog vaccination coverage to 7% or 13% in the next five years, rabies deaths could be reduced by 70% or 88%, respectively, suggesting a slight improvement in dog vaccination coverage yields substantial health benefits [36]. In this sense, it is practical to improve dog vaccination coverage gradually in some areas of South China (e.g., Guangxi), from the current 19.1% to the national average level of 40%, and further to the aim of 70%. Moreover, studies in the Philippines have indicated that dog population management programmes, such as dog spaying/neutering and shelters, can effectively manage limited resources and reduce human rabies cases by increasing dog vaccination coverage rates [37]. Further research is necessary for China to assess the impact of programmes for dog population management, specifically in rural areas and under the livestock/veterinary sector supervision.

The WHO proposed the role of IBCM in evaluating the risk of rabies exposure, which formally engages medical and veterinary multi-sectors from the "One Health" viewpoint; however, it has not been implemented in China at all [10]. The purpose of IBCM in other rabies-epidemic countries, such as Tanzania and Haiti, is to improve PEP compliance to reduce the rabies burden [38, 39]. For example, in Haiti, the probabilities of health-seeking, receiving PEP treatment, and completing the regimen were 54%, less than 50%, and less than 40%, respectively [38]. In contrast, the corresponding probabilities in China were 85%, 99%, and 91%, respectively [21, 31]. The WHO recommended that countries also avoid overuse of PEP, especially when successful control activities cause rabies to decline [28]. Since adequate PEP access has been provided in China [9], the primary aim of IBCM is expected to reduce the costs of unnecessary use of PEP; thus, the strategies combined with IBCM in our study dominate others in terms of cost-effectiveness. Nevertheless, the success of IBCM relies on close collaboration between the health and livestock/veterinary sectors to ensure timely dog bite reporting and a skilled workforce to assess animals and make diagnoses [40]. Cost estimates for IBCM should include factors such as the number of staff needed and their working hours [38]. Considering regional disparities in the rabies epidemic and dog vaccination coverage, a pilot study on implementing IBCM is needed in China.

To the best of our knowledge, this is the first study that used the updated national data to predict the future burden of human rabies and assess the cost-effectiveness of comprehensive rabies-control strategies in China. Moreover, we considered the regional disparities in the rabies epidemic and the economic level in China to inform the evidence-based practical implementation of these strategies for rabies elimination by areas. Nevertheless, as with other modelling studies, our study also has limitations that warrant consideration when interpreting the results. First, some of our assumptions in the model may vary over time; for example, the natural growth rate of the human population will change with time, while we assumed that the values of these parameters were constant for simplicity. In addition, we used similar methods and assumptions to the WHO Rabies Modelling Consortium study for international comparisons. Second, the parameters used in analyses were mainly from official data confirmed in the national rabies surveillance system or published results in other countries; for example, the estimation of the bitten incidence of rabid dogs was from Tanzania [6]. However, our intention was to highlight the cost-effective strategies that could make the elimination goal achievable, rather than just making predictions on the precise elimination time. In this regard, various sensitivity analyses allowed a comprehensive approach to the assessment of the robustness of our conclusions. Finally, with the same methods used in the WHO Rabies Modelling Consortium study, some region-specific parameters were obtained from the national-level values due to a lack of available data. Thus, these homogenous average estimates may inevitably result in underestimating regional disparities. Further investigations on region-specific surveys in more areas of China are therefore required.

## Conclusions

In summary, the burden of Chinese rabies death remains stable in the next decade under the status quo; however, scaling up dog vaccination instead of expanding PEP access is crucial to achieving the elimination goal in China as early as 2033. The combined strategy of mass dog vaccination and IBCM could substantially reduce the rabies burden and costs of unnecessary PEP use. Eliminating dog-mediated rabies in China cost-effectively requires the "One Health" approach to prioritize progress in the livestock/veterinary sectors for widespread rural dog vaccination. The successful implementation of IBCM also calls for collaboration between the health and livestock/veterinary sectors. It would be the most cost-effective strategy recommended for interrupting rabies transmission in China, thus eventually contributing to the global plan of ending dog-mediated human rabies death.

## Supplementary Information

**Additional file 1: Statistical Analysis Plan.** A health economic analysis plan. **CHEERS 2022 Checklist.** Our study followed the updated Consolidated Health Economic Evaluation Reporting Standards 2022 (CHEERS 2022) checklist. **Fig. S1** Epidemiology of dog-mediated human rabid deaths of China during 2011–2020. **Fig. S2** Diagram of the decision tree model. **Fig. S3** Numbers of reported dog-mediated human rabid deaths in China during 2011–2020. **Fig. S4** The trends of the number of dog-mediated human rabies deaths, the number of people receiving the PEP, and the vaccine vials used in different scenarios with IBCM from 2023 to 2035 in China. **Fig. S5** One-way sensitivity analyses. **Fig. S6** Probabilistic sensitivity analyses of the total rabies deaths, DALYs, cost per death averted and cost per DALY averted during 2024–2035 in China. **Table S1.** The description of the scenarios in the STRATEGIC study. **Table S2.** The nation-level parameters under the scenario of the status quo (Scenario 1) in China. **Table S3.** The parameter values under scenarios using different strategies for rabies control in China. **Table S4.** The region-specific parameters under the scenario of the status quo (Scenario 1). **Table S5.** The life table for the estimation of life expectancy. **Table S6.** The predicted number of dog-mediated human rabies deaths in different scenarios with IBCM during 2023–2035 in China by regions. **Table S7.** The incremental cost-effectiveness ratio per death prevented by different strategies compared with the status quo in China during 2024–2035. **Table S8.** The ICER per death prevented by different strategies compared with the status quo during 2024–2035 in Shandong (East China). **Table S9.** The ICER per death prevented by different strategies compared with the status quo during 2024–2035 in Hunan (Central China). **Table S10.** The ICER per death prevented by different strategies compared with the status quo during 2024–2035 in Tianjin (North China). **Table S11.** The ICER per death prevented by different strategies compared with the status quo during 2024–2035 in Guangxi (South China). **Table S12.** The ICER per death prevented by different strategies compared with the status quo during 2024–2035 in Shaanxi (Northwest China). **Table S13.** The ICER per death prevented by different strategies compared with the status quo during 2024–2035 in Guizhou (Southwest China).

## Data Availability

All data relevant to the study are included in the article and Additional files. The STRATEGIC study model source code can be found in the GitHub repository (https://github.com/pkuepi/STRATEGIC).

## Abbreviations

WHO: World Health Organization
PEP: Post-exposure prophylaxis
IBCM: Integrated bite case management
STRATEGIC: Strategies to inTerrupt RAbies Transmission for the Elimination Goal by 2030 In China
China CDC: Chinese Center for Disease Control and Prevention
NHRS: National Human Rabies Surveillance
CHEERS: Consolidated Health Economic Evaluation Reporting Standards
DALYs: Disability-adjusted life-years
ICER: Incremental cost-effectiveness ratio
GDP: Gross domestic product
RIG: Rabies immunoglobulin
PSA: Probabilistic sensitivity analyses
UI: Uncertainty interval
