# Supplementary material for: Strategies to inTerrupt RAbies Transmission for the Elimination Goal by 2030 In China (STRATEGIC): a modelling study
Source: BMC Med. 2023 Mar 16;21:100. doi: 10.1186/s12916-023-02821-x (PMC10022085; doi:10.1186/s12916-023-02821-x)
Supplement: Supplementary file 1 — Additional file 1: Statistical Analysis Plan. A health economic analysis plan. CHEERS 2022 Checklist. Our study followed the updated Consolidated Health Economic Evaluation Reporting Standards 2022 (CHEERS 2022) checklist. Fig. S1 Epidemiology of dog-mediated human rabid deaths of China during 2011–2020. Fig. S2 Diagram of the decision tree model. Fig. S3 Numbers of reported dog-mediated human rabid deaths in China during 2011–2020. Fig. S4 The trends of the number of dog-mediated human rabies deaths, the number of people receiving the PEP, and the vaccine vials used in different scenarios with IBCM from 2023 to 2035 in China. Fig. S5 One-way sensitivity analyses. Fig. S6 Probabilistic sensitivity analyses of the total rabies deaths, DALYs, cost per death averted and cost per DALY averted during 2024–2035 in China. Table S1. The description of the scenarios in the STRATEGIC study. Table S2. The nation-level parameters under the scenario of the status quo (Scenario 1) in China. Table S3. The parameter values under scenarios using different strategies for rabies control in China. Table S4. The region-specific parameters under the scenario of the status quo (Scenario 1). Table S5. The life table for the estimation of life expectancy. Table S6. The predicted number of dog-mediated human rabies deaths in different scenarios with IBCM during 2023–2035 in China by regions. Table S7. The incremental cost-effectiveness ratio per death prevented by different strategies compared with the status quo in China during 2024–2035. Table S8. The ICER per death prevented by different strategies compared with the status quo during 2024–2035 in Shandong (East China). Table S9. The ICER per death prevented by different strategies compared with the status quo during 2024–2035 in Hunan (Central China). Table S10. The ICER per death prevented by different strategies compared with the status quo during 2024–2035 in Tianjin (North China). Table S11. The ICER per death prevented by different s [file 12916_2023_2821_MOESM1_ESM.docx]

**Strategies to inTerrupt RAbies Transmission for the Elimination Goal by 2030 In China (STRATEGIC): a modelling study**

**Additional file 1**

[A. Additional Methods 1](#_Toc127320689)

[Statistical Analysis Plan 1](#_Toc127320690)

[CHEERS 2022 Checklist 5](#_Toc127320691)

[B. Figures 8](#_Toc127320692)

[Fig. S1 Epidemiology of dog-mediated human rabid deaths of China during 2011-2020 8](#_Toc127320693)

[Fig. S2 Diagram of the decision tree model 9](#_Toc127320694)

[Fig. S3 Numbers of reported dog-mediated human rabid deaths in China during 2011-2020 10](#_Toc127320695)

[Fig. S4 The trends of the number of dog-mediated human rabies deaths, the number of people receiving the PEP, and the vaccine vials used in different scenarios with IBCM from 2023 to 2035 in China 12](#_Toc127320696)

[Fig. S5 One-way sensitivity analyses 13](#_Toc127320697)

[Fig. S6 Probabilistic sensitivity analyses of the total rabies deaths, DALYs, cost per death averted and cost per DALY averted during 2024-2035 in China 14](#_Toc127320698)

[C. Tables 15](#_Toc127320699)

[Table S1. The description of the scenarios in the STRATEGIC study 15](#_Toc127320700)

[Table S2. The nation-level parameters under the scenario of the status quo (*Scenario 1*) in China 16](#_Toc127320701)

[Table S3. The parameter values under scenarios using different strategies for rabies control in China 19](#_Toc127320702)

[Table S4. The region-specific parameters under the scenario of the status quo (*Scenario 1*) 20](#_Toc127320703)

[Table S5. The life table for the estimation of life expectancy 21](#_Toc127320704)

[Table S6. The predicted number of dog-mediated human rabies deaths in different scenarios with IBCM during 2023-2035 in China by regions 23](#_Toc127320705)

[Table S7. The incremental cost-effectiveness ratio per death prevented by different strategies compared with the status quo in China during 2024-2035 24](#_Toc127320706)

[Table S8. The ICER per death prevented by different strategies compared with the status quo during 2024-2035 in Shandong (East China) 25](#_Toc127320707)

[Table S9. The ICER per death prevented by different strategies compared with the status quo during 2024-2035 in Hunan (Central China) 26](#_Toc127320708)

[Table S10. The ICER per death prevented by different strategies compared with the status quo during 2024-2035 in Tianjin (North China) 27](#_Toc127320709)

[Table S11. The ICER per death prevented by different strategies compared with the status quo during 2024-2035 in Guangxi (South China) 28](#_Toc127320710)

[Table S12. The ICER per death prevented by different strategies compared with the status quo during 2024-2035 in Shaanxi (Northwest China) 29](#_Toc127320711)

[Table S13. The ICER per death prevented by different strategies compared with the status quo during 2024-2035 in Guizhou (Southwest China) 30](#_Toc127320712)

# A. Additional Methods

## Statistical Analysis Plan

**Statistical Analysis Plan**

March 17^th^, 2022

1. **Title**

- Strategies to inTerrupt RAbies Transmission for the Elimination Goal by 2030 In China (STRATEGIC): a modelling study

1. **Objective**

- To predict the trends of human deaths from dog-mediated rabies during 2023-2035 in China;
- To assess the cost-effectiveness of combination strategies of expanding post-exposure prophylaxis (PEP), mass dog vaccination, and integrated bite case management (IBCM) for interrupting rabies transmission during 2023-2035 in China;
- To investigate the potential regional disparities in the effectiveness/cost-effectiveness of rabies control strategies during 2023-2035 in different areas of China.

1. **Data**

- Surveillance and survey data on rabies exposure, health care activities, and costs are provided by the National Human Rabies Surveillance (NHRS) System in the Chinese Center for Disease Control (China CDC) to estimate health and economic outcomes.
- Population data of the age-specific distributions in 2020 obtained from the National Bureau of Statistics of China, all-cause mortality data from the Global Burden of Disease study in 2019 and rabies mortality data from the National Human Rabies Surveillance (NHRS) System in China CDC are used to calculate the disability-adjusted life-years (DALYs).
- Region-specific data are collected from provincial surveillance points, including Shandong (East China), Hunan (Central China), Tianjin (North China), Guangxi (South China), Shaanxi (Northwest China) and Guizhou (Southwest China), to explore regional disparities.
- Some parameters are adopted from the World Health Organization (WHO) Rabies Modelling Consortium study and other published literature.

1. **Study design**

A decision-analytic modelling study.

1. **Outcomes**

The outcome to be simulated is the dog-mediated human rabies death.

1. **Methods**

- Human rabies dynamics and costs are simulated in a decision tree model published by the WHO Rabies Modelling Consortium study.^1^
- This study will comply with the updated Consolidated Health Economic Evaluation Reporting Standards (CHEERS) 2022 checklist.^2^
- All statistical analyses will be performed using R version 4.0.5. (R Foundation for Statistical Computing, Vienna, Austria. <https://www.r-project.org/>).

1. **Parameters**

- The number of human population is estimated by data from the National Bureau of Statistics of China. The human-to-dog ratio is used to estimate the number of dogs.
- Parameters related to rabies exposure are used to estimate the number of people bitten by dogs every year, including dog rabies incidence (*P*_rabid_), probability of rabid dog bites (*P*_bite1_), probability of infection after being bitten by a rabid dog (*P*_infect_), and probability of being bitten by a healthy dog (*P*_bite2_).
- Parameters related to health care activities are used to estimate the human deaths caused by dog-mediated rabies, including the probability of seeking medical care after being bitten (*P*_seek_), probability of receiving PEP treatment (*P*_receive1_), probability of completing PEP treatment (*P*_complete_), the protective effect of complete PEP treatment (*P*_prevent1_) and the protective effect of incomplete PEP treatment (*P*_prevent2_).
- The age-specific life expectancy calculated by a life table is used to estimate DALYs.

The main parameters of the model are listed in **Table 1**.

**Table 1 The parameters in the model**

| Parameter | Description | Value | Source |
| --- | --- | --- | --- |
| *P*_rabid_ | Rabid incidence in dogs |  |  |
| *P*_infect_ | Probability of infection after exposure without PEP |  |  |
| *P*_bite1_ | Probability of being bitten by a rabid dog |  |  |
| *P*_bite2_ | Probability of being bitten by a healthy dog |  |  |
| *P*_seek_ | Probability of seeking health care after being bitten |  |  |
| *P*_receive1_ | Probability of receiving PEP vaccine if treatment sought |  |  |
| *P*_complete_ | Probability of completing PEP vaccination regimen |  |  |
| *P*_prevent1_ | Probability of being alive if complete PEP prevents rabies |  |  |
| *P*_prevent2_ | Probability of being alive if incomplete vaccination prevents rabies |  |  |

1. **Primary analyses**

**Scenarios and assumptions**

Eight scenarios are simulated to evaluate different strategies for improved post-exposure prophylaxis (PEP) access, mass dog vaccination, and patient risk assessments with integrated bite case management (IBCM), as follows:

1. *Status quo*. In this scenario, rabies prevention is performed based on the current practice in China.
2. *Increased PEP access*. In this scenario, we assume that the health care provider will cover the cost of PEP treatment. Consequently, the probability of health-seeking, receiving PEP treatment, and completing PEP treatment will increase at a constant rate annually.
3. *Scaling up mass dog vaccination coverage*. In this scenario, we assume that the number of rabid dogs will decrease as mass vaccination coverage increases.^3,4^ There are two sub-scenarios, (3a) increased mass dog vaccination coverage based on *scenario 1,* and (3b) increased mass dog vaccination coverage based on *scenario 2*.
4. *Use of IBCM*. There are four sub-scenarios, including (4a) IBCM based on *scenario 1*; (4b) IBCM based on *scenario 2*; (4c) IBCM based on *scenario 3a*; and (4d) IBCM based on *scenario 3b.*

We will measure health benefits from human rabies deaths and DALYs and only consider the direct costs. Each scenario will be simulated 1000 times to obtain the 95% uncertainty interval (UI).

**The main statistics to be reported**

Annual human rabies deaths, human rabies deaths averted, vaccine vials used, and costs, with 95% UI under different scenarios.

**Subgroup analyses**

We will examine regional disparities using the data collected from the six diverse areas of China.

**Discount rate and exchange rate**

All costs will be converted to US dollars at the exchange rate in 2020, with a discount rate of 3%.

1. **Sensitivity analyses**

The one-way sensitivity analyses will consider the uncertainty of the following parameters:

- incidence of rabid dog bites per person annually
- incidence of non-rabid dog bites per person annually
- probability of developing rabies with exposure
- probability of preventing rabies by complete or incomplete PEP treatment

1. **Proposed Main Tables & Figures**

*Table 1* The descriptions of scenarios in the study

- Descriptions of scenarios to be simulated

*Table 2* The parameters in the decision tree model

- Parameter values and their sources in the decision tree model

*Table 3* The cumulative number of dog-mediated human rabies deaths from 2023 to 2035 in China by areas

- Main results of human rabies deaths, human rabies deaths averted, vaccine vials used and costs over the years using different strategies.

*Figure 1* The structure diagram of the decision tree model

*Figure 2* Map of the burden of dog-mediated human rabid deaths in China

*Figure 3* Trends of the number of dog-mediated human rabies deaths, the number of persons receiving the PEP, and the vaccine vials used during 2023-2035 in China

*Figure 4* The cost-effectiveness plane for different strategies in China by areas

*Figure 5* Results of the one-way sensitivity analyses

1. **References**

1. World Health Organization (WHO) Rabies Modelling Consortium. The potential effect of improved provision of rabies post-exposure prophylaxis in Gavi-eligible countries: a modelling study. Lancet Infect Dis. 2019; 19(1):102-111.

2. Husereau D, Drummond M, Augustovski F, de Bekker-Grob E, Briggs AH, Carswell C, et al. Consolidated Health Economic Evaluation Reporting Standards 2022 (CHEERS 2022) statement: updated reporting guidance for health economic evaluations. BMJ. 2022; 376:e067975.

3. Hampson K, Dushoff J, Cleaveland S, Haydon DT, Kaare M, Packer C, et al. Transmission Dynamics and Prospects for the Elimination of Canine Rabies. PLoS Biol. 2009; 7(3):e1000053.

4. Townsend SE, Sumantra IP, Pudjiatmoko, Bagus GN, Brum E, Cleaveland S, et al. Designing Programs for Eliminating Canine Rabies from Islands: Bali, Indonesia as a Case Study. PLoS Negl Trop Dis. 2013; 7(8):e2372.

## CHEERS 2022 Checklist

**Consolidated Health Economic Evaluation Reporting Standards 2022 (CHEERS 2022)**

| **Section/topic** | **Guidance for reporting** | **Reported in section** |
| --- | --- | --- |
| **Title** | | |
|  | Identify the study as an economic evaluation and specify the interventions being compared. | Title |
| **Abstract** | | |
|  | Provide a structured summary that highlights context, key methods, results, and alternative analyses. | Abstract |
| **Introduction** | | |
| Background and objectives | Give the context for the study, the study question, and its practical relevance for decision making in policy or practice. | Background |
| **Methods** | | |
| Health economic analysis plan | Indicate whether a health economic analysis plan was developed and where available. | Additional file 1: Statistical Analysis Plan |
| Study population | Describe characteristics of the study population (such as age range, demographics, socioeconomic, or clinical characteristics). | Methods, paragraph 1 |
| Setting and location | Provide relevant contextual information that may influence findings. | Methods, paragraph 1 |
| Comparators | Describe the interventions or strategies being compared and why chosen. | Methods, paragraph 2 |
| Perspective | State the perspective(s) adopted by the study and why chosen. | Methods, paragraph 4 |
| Time horizon | State the time horizon for the study and why appropriate. | Methods, paragraph 4 |
| Discount rate | Report the discount rate(s) and reason chosen. | Methods, paragraph 4 |
| Selection of outcomes | Describe what outcomes were used as the measure(s) of benefit(s) and harm(s). | Methods, paragraph 4 |
| Measurement of outcomes | Describe how outcomes used to capture benefit(s) and harm(s) were measured. | Methods, paragraph 5-12 |
| Valuation of outcomes | Describe the population and methods used to measure and value outcomes. | Methods, paragraph 3 |
| Measurement and valuation of resources and costs | Describe how costs were valued. | Methods, paragraph 12 |
| Currency, price date, and conversion | Report the dates of the estimated resource quantities and unit costs, plus the currency and year of conversion. | Methods, paragraph 12 |
| Rationale and description of model | If modelling is used, describe in detail and why used. Report if the model is publicly available and where it can be accessed. | Methods, paragraph 3 |
| Analytics and assumptions | Describe any methods for analysing or statistically transforming data, any extrapolation methods, and approaches for validating any model used. | Methods |
| Characterising heterogeneity | Describe any methods used for estimating how the results of the study vary for subgroups. | Methods, paragraph 1 |
| Characterising distributional effects | Describe how impacts are distributed across different individuals or adjustments made to reflect priority populations. | Methods, paragraph 1 |
| Characterising uncertainty | Describe methods to characterise any sources of uncertainty in the analysis. | Methods, paragraph 13 |
| Approach to engagement with patients and others affected by the study | Describe any approaches to engage patients or service recipients, the general public, communities, or stakeholders (such as clinicians or payers) in the design of the study. | NA |
| **Results** | | |
| Study parameters | Report all analytic inputs (such as values, ranges, references) including uncertainty or distributional assumptions. | Additional file 1: Tables S2-S4; Methods, paragraph 5-12 |
| Summary of main results | Report the mean values for the main categories of costs and outcomes of interest and summarise them in the most appropriate overall measure. | Results |
| Effect of uncertainty | Describe how uncertainty about analytic judgments, inputs, or projections affect findings. Report the effect of choice of discount rate and time horizon, if applicable. | Results, paragraph 6-7 |
| Effect of engagement with patients and others affected by the study | Report on any difference patient/service recipient, general public, community, or stakeholder involvement made to the approach or findings of the study | NA |
| **Discussion** | | |
| Study findings, limitations, generalisability, and current knowledge | Report key findings, limitations, ethical or equity considerations not captured, and how these could affect patients, policy, or practice. | Discussion |
| **Other relevant information** | | |
| Source of funding | Describe how the study was funded and any role of the funder in the identification, design, conduct, and reporting of the analysis | Funding |
| Conflicts of interest | Report authors conflicts of interest according to journal or International Committee of Medical Journal Editors requirements. | Competing interests |

# B. Figures


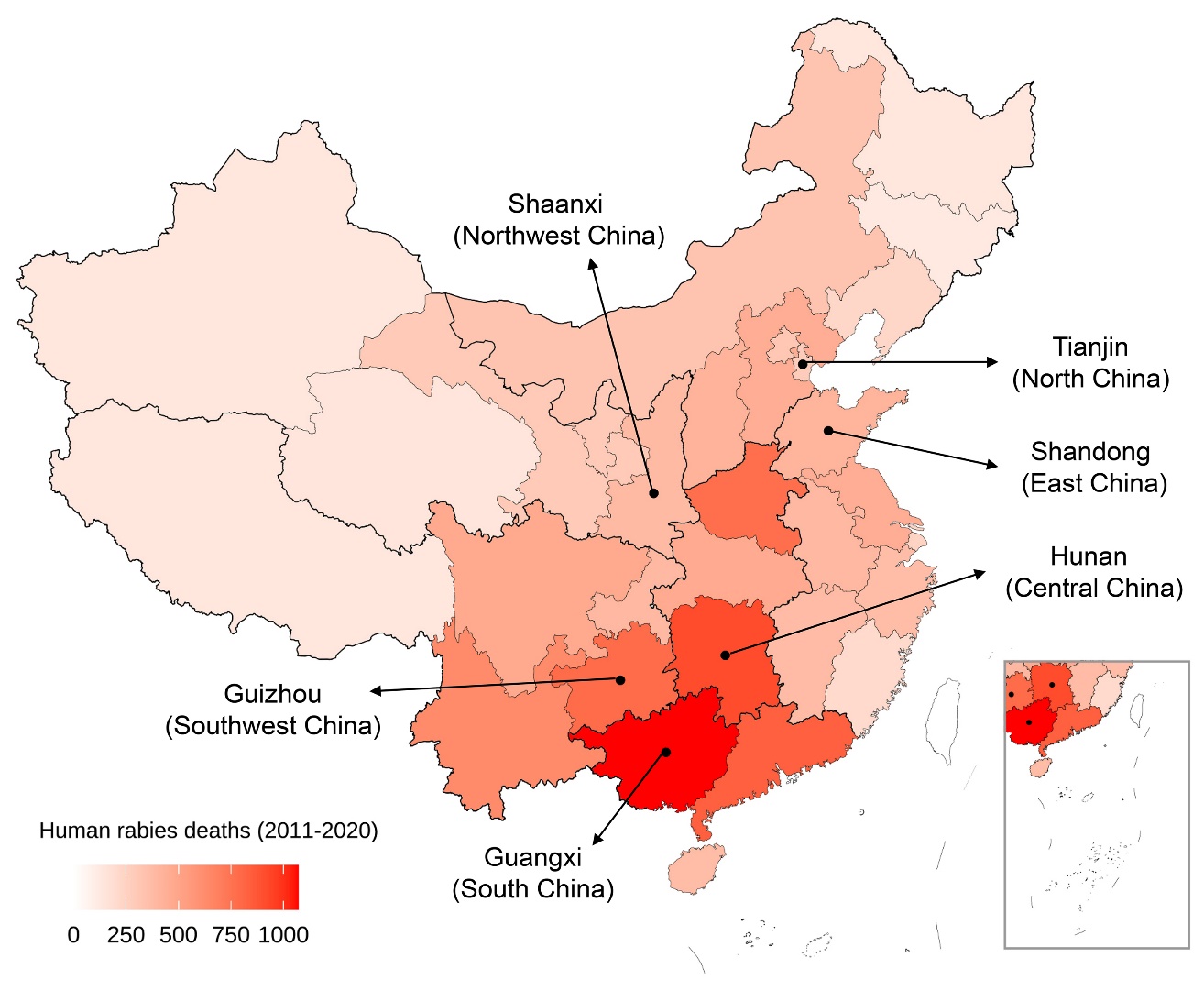


## Fig. S1 Epidemiology of dog-mediated human rabid deaths of China during 2011-2020


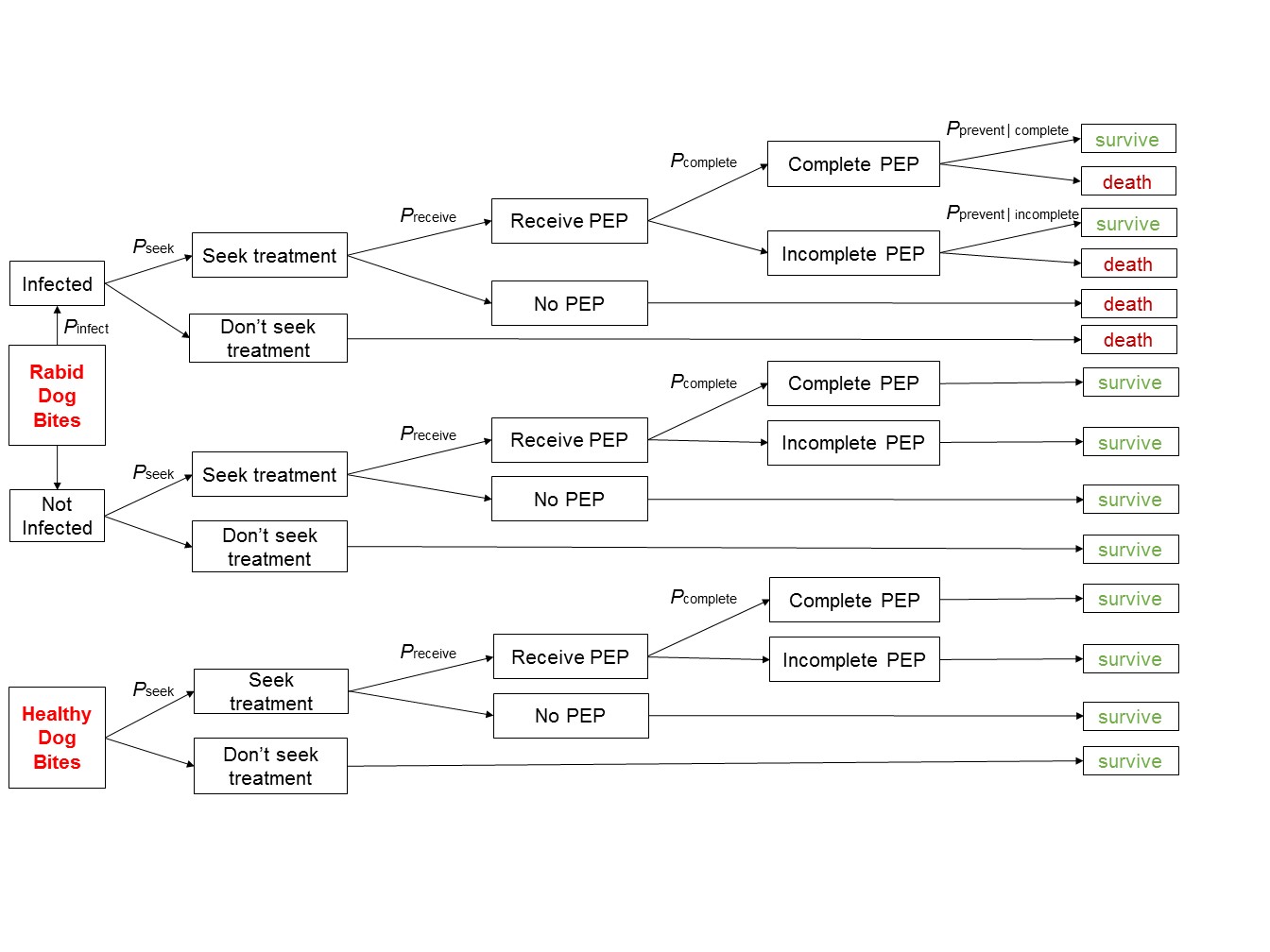


## Fig. S2 Diagram of the decision tree model

The decision tree model was used to obtain health outcomes and costs by simulating a person's behaviour of seeking medical care after being bitten by a dog, using parameters from published literature and data from the national human rabies surveillance system. ***P***infect, probability of developing rabies without any intervention; ***P***seek, probability of seeking medical care after being bitten; ***P***receive, probability of receiving PEP if treatment sought; ***P***complete, probability of completing PEP regimen if PEP received; ***P***prevent, probability of avoiding rabies given a complete or incomplete PEP treatment. PEP, post-exposure prophylaxis.***
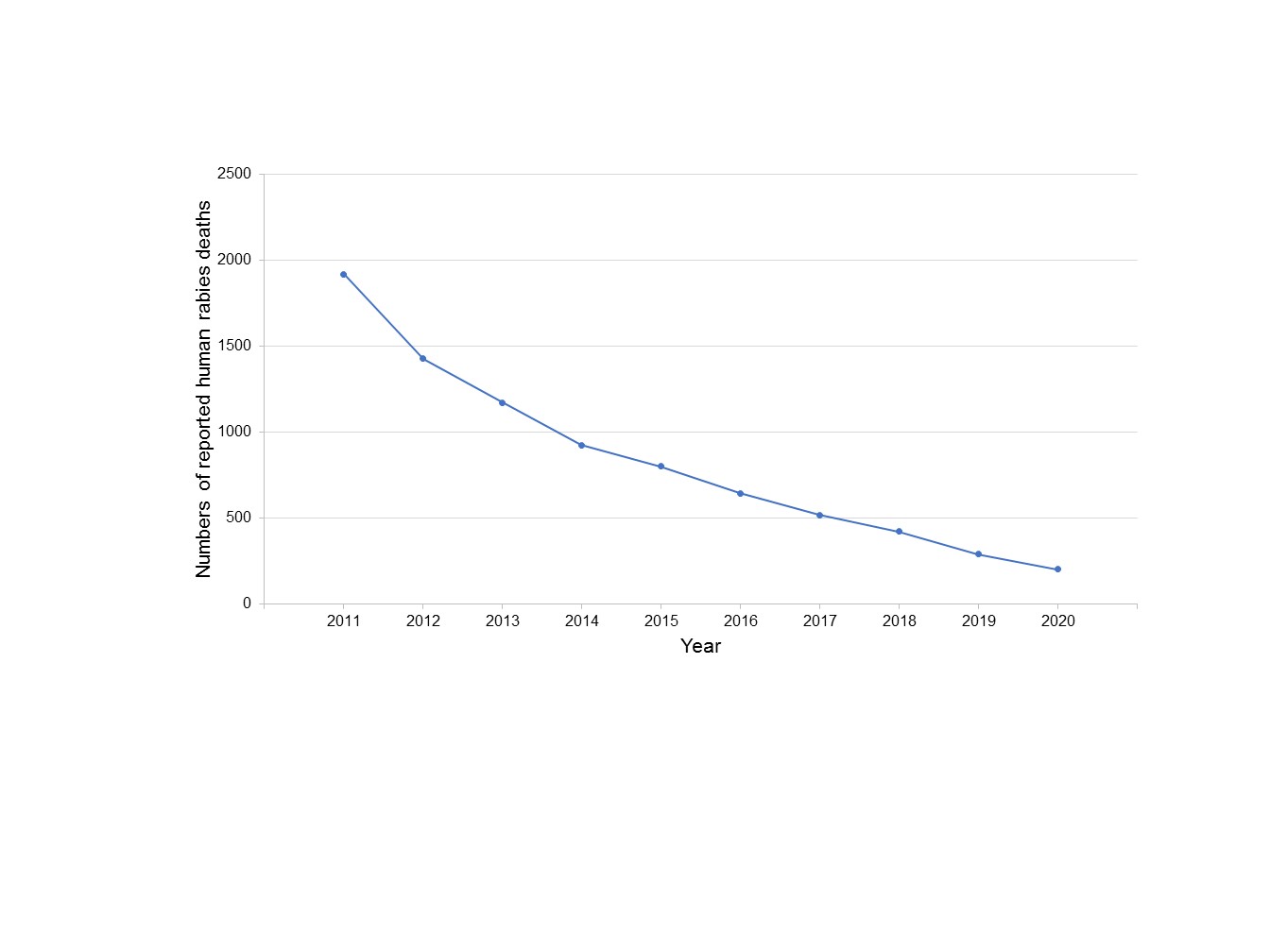
***

## Fig. S3 Numbers of reported dog-mediated human rabid deaths in China during 2011-2020

***
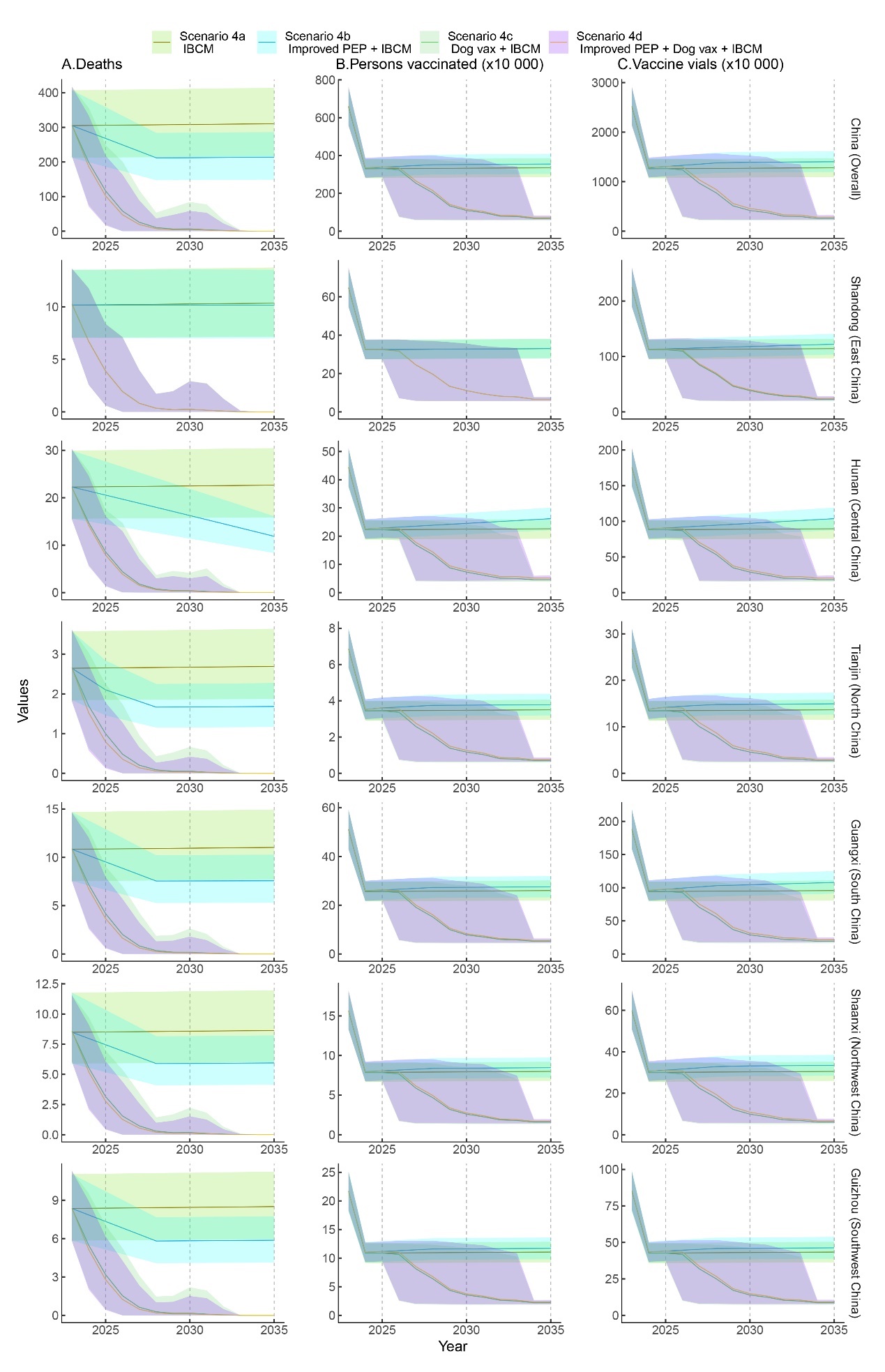
***

## Fig. S4 The trends of the number of dog-mediated human rabies deaths, the number of people receiving the PEP, and the vaccine vials used in different scenarios with IBCM from 2023 to 2035 in China

Scenario 4a, Status Quo + IBCM; Scenario 4b, Improved PEP + IBCM; Scenario 4c, Improved mass dog vaccination + IBCM; Scenario 4d, Improved PEP + improved mass dog vaccination +IBCM. PEP, post-exposure prophylaxis; IBCM, integrated bite case management.

**
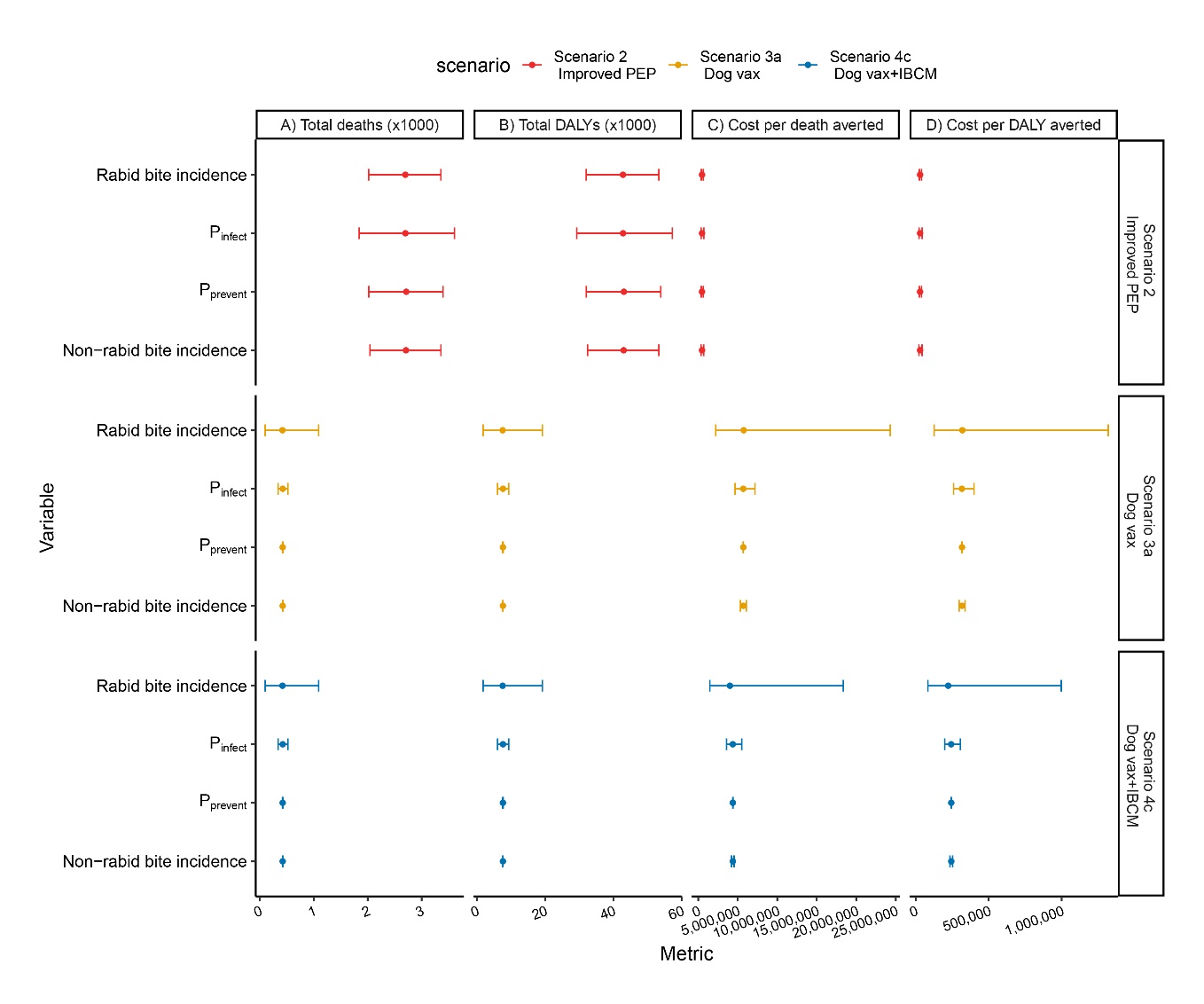
**

## Fig. S5 One-way sensitivity analyses

***P***_infect_, probability of developing rabies without any intervention; ***P***_prevent_, probability of avoiding rabies given an incomplete PEP treatment. PEP, post-exposure prophylaxis; Dog vax, dog vaccination; IBCM, integrated bite case management.


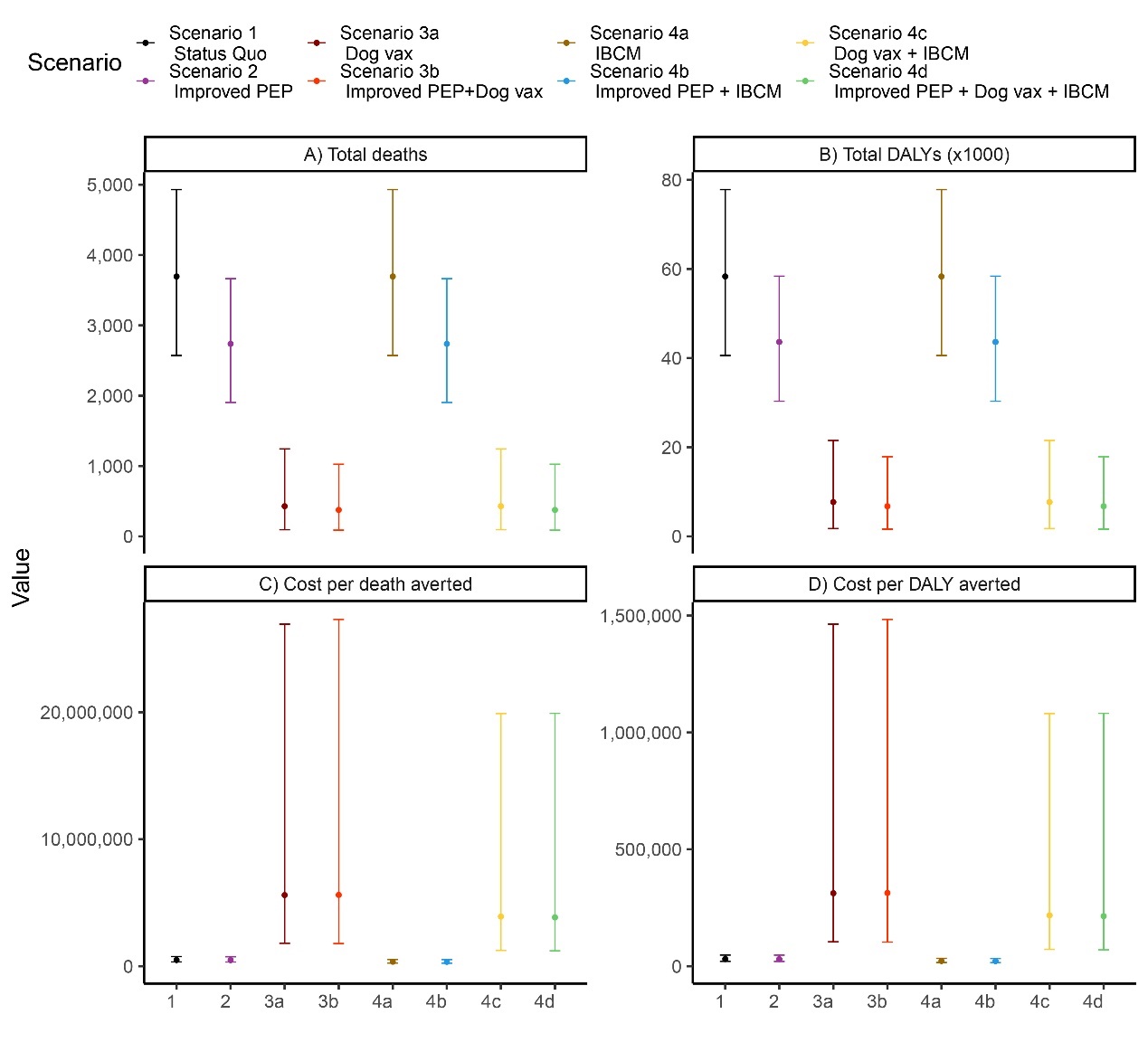


## Fig. S6 Probabilistic sensitivity analyses of the total rabies deaths, DALYs, cost per death averted and cost per DALY averted during 2024-2035 in China

DALYs, disability-adjusted life-years; PEP, post-exposure prophylaxis; IBCM, integrated bite case management; Dog vax, dog vaccination.

# C. Tables

## Table S1. The description of the scenarios in the STRATEGIC study

| **Scenario** | **Description of the scenario** |
| --- | --- |
| 1. *Status quo* | Rabies prevention is performed according to the current practice in China without IBCM as usual, i.e., victims bitten by dogs and seek PEP treatment in clinics and paid by themselves, while mass dog vaccination remains low, below 70% |
| 1. *Improved PEP access* | Increased PEP access based on the *scenario (1)* We assumed that basic health insurance would cover the cost of PEP treatment to increase the probability of health-seeking, receiving and completing PEP treatment. |
| 1. *Scaling up mass dog vaccination coverage* | We assumed that the number of rabid dogs would decrease as dog vaccination coverage increased to 70% (especially in rural areas by livestock/veterinary sector), as recommended by the WHO. Two sub-scenarios were included: |
|  | (3a) Increased mass dog vaccination coverage based on the *scenario (1)* |
|  | (3b) Increased mass dog vaccination coverage based on the *scenario (2)* |
| 1. *Use of the IBCM* | We evaluated the impact of the IBCM approach, where the health sector and livestock/veterinary sector collaborate for the risk assessment of patients bitten by dogs. Four sub-scenarios were considered: |
|  | (4a) Use of the IBCM based on the *scenario (1)* |
|  | (4b) Use of the IBCM based on the *scenario (2)* |
|  | (4c) Use of the IBCM based on the *scenario (3a)* |
|  | (4d) Use of the IBCM based on the *scenario (3b)* |

PEP, post-exposure prophylaxis; IBCM, integrated bite case management.

## Table S2. The nation-level parameters under the scenario of the status quo (*Scenario 1*) in China

|  | **Explanation** | **Value (95% CrI of posterior where applicable)** | **Distribution** | **Reference or sources** | |
| --- | --- | --- | --- | --- | --- |
| ***Parameters related to the rabies exposure*** | | | | | |
| n_man in year 2020_^*^ | The number of human population in 2020 | 1,412 million | fixed | 2020 China population census^[1]^ | |
| n_dog in year 2020_^**^ | The number of dog population in 2020 | 100 million | fixed | Published literature^[2]^ | |
| n_bitten by all dog_ | The number of human population bitten by all dogs in 2020 | 7.78 million | fixed | NHRS system and Published literature^[2]^ | |
| *P*_bite\|rabid dog_ | The average number of bites per rabid dog | 0.38 | fixed | Published literature^[3]^ | |
| *P*_rabid\|dog_ | Rabies incidence in dogs | 0.0003(0.000225-0.000375) | normal | Expert consultation based on data from the first Chinese Rabies Surveillance Plan in animal populations during 2004-2018^[4]^ |  |
| ***Parameters related to the health care activities*** | | | | |  |
| *P*_seek_ | Probability of seeking medical care after being bitten | 0.85 | fixed | NHRS system and published literature^[5-6]^ | |
| *P*_receive1_ | Probability of receiving PEP if treatment sought | 0.99 | fixed | NHRS system and published literature^[7-9]^ | |
| *P*_complete_ | Probability of completing PEP regimen if PEP received | 0.91 | fixed | NHRS system and published literature^[7-10]^ | |
| *P*_receive2_ | Probability of receiving Rabies immunoglobulin after being bitten | 0.17 | fixed | NHRS system and published literature^[7-10]^ | |
| *P*_infect_ | Probability of developing rabies without any intervention | 0.16 (0.13-0.20) | binomial | Published literature^[11]^ | |
| *P*_prevent\|rig_ | Probability of avoiding rabies given an RIG injection | 1 | fixed | Published literature^[12]^ | |
| *P*_prevent\|complete_ | Probability of avoiding rabies given a complete PEP | 1.00 (0.99-1.00) | binomial | Published literature^[11]^ | |
| *P*_prevent\|incomplete_ | Probability of avoiding rabies given an incomplete PEP | 0.99 (0.98-0.99) | binomial | Published literature^[11]^ | |
| ***Parameters related to the costs (in US dollars)*** | | | | | |
| C_vaccine_ | PEP vaccine price, per dose | 8.6962 | fixed | NHRS system and published literature^[13-14]^ | |
| C_RIG_ | Rabies immunoglobulin price, per dose | 144.9359 | fixed | NHRS system and published literature^[13-14]^ | |
| C_vaccine injection_ | PEP vaccine injection price, per visit | 3.1175 | fixed | NHRS system | |
| C_RIG injection_ | Rabies immunoglobulin injection price, per visit | 3.7566 | fixed | NHRS system | |
| C_wound cleaning_ | wound cleaning price, per visit | 13.7447 | fixed | NHRS system | |
| C_registration (first visit)_ | registration fee for the first visit | 1.0813 | fixed | NHRS system | |
| C_other cost (first visit)_ | other cost of the first visit | 0.3629 | fixed | NHRS system | |
| C_registration (follow up)_ | registration fee for the follow-up | 0.3408 | fixed | NHRS system | |
| C_dog vaccine_ | annual dog vaccination fee | 11.33 | fixed | WHO document^[15]^ | |

PEP, post-exposure prophylaxis. NHRS, National Human Rabies Surveillance

*The number of human population beyond 2020 was simulated using the birth rate and mortality rate of 0.852% and 0.707% from National Bureau of Statistics of China.

**The number of dog population beyond 2020 was estimated by the number of human population in each year and a constant value (14) of the human-to-dog ratio.

1. National Bureau of Statistics of China. National data. https://data.stats.gov.cn/. Accessed 7 March 2023.
2. Yin W, F. Fu Z, F. Gao G. Progress and Prospects of Dog-Mediated Rabies Elimination in China. China CDC Wkly. 2021; 3(39):831-834.
3. Hampson K, Abela-Ridder B, Brunker K, Bucheli ST, Carvalho M, Caldas E, et al. Surveillance to establish elimination of transmission and freedom from dog-mediated rabies. BioRxiv. 2016; https:// doi. org/ 10. 1101/ 096883. Accessed 7 March 2023.
4. Feng Y, Wang Y, Xu W, Tu Z, Liu T, Huo M, et al. Animal Rabies Surveillance, China, 2004-2018. Emerg Infect Dis. 2020; 26(12):2825-2834.
5. Yang H, Li B, Luo K, Sun Q, Zhang H, Zhao S, et al. Investigation on rabies exposure and its influencing factors in population in rural areas of Hunan Province. J Trop Dis Parasitol. 2021; 19(1):19-24. (in Chinese)
6. Zhu M, Mu D, Chen Q, Chen N, Zhang Y, Yin W, et al. Awareness Towards Rabies and Exposure Rate and Treatment of Dog-Bite Injuries Among Rural Residents - Guangxi Zhuang Autonomous Region, China, 2021. China CDC Wkly. 2021; 3(53):1139-1142.
7. Liu Y, Li L, Lyu J, Su C. Epidemiological Study of Outpatients in Rabies Post-Exposure Prophylaxis Clinics - Tianjin Municipality, China, 2020. China CDC Wkly. 2021; 3(39):822-824.
8. Liu J, Duo L, Tao X, Zhu W. Epidemiological characteristics of human rabies in China, 2017. Chin J Epidemiol. 2019; 40(5):526-530. (in Chinese)
9. Yin C, Zhou H, Wu H, Tao X, Rayner S, Wang S, et al. Analysis on factors related to rabies epidemic in China from 2007-2011. Virol Sin. 2012; 27(2):132-143.
10. Liu M, Liu Z, Tao X, Zhu W. Epidemiological characteristics of rabies in China, 2020. Disease Surveillance. 2022; 37(5):609-612. (in Chinese)
11. Changalucha J, Steenson R, Grieve E, Cleaveland S, Lembo T, Lushasi K, et al. The need to improve access to rabies post-exposure vaccines: Lessons from Tanzania. Vaccine. 2019; 37(Suppl 1):A45-A53.
12. World Health Organization (WHO) Rabies Modelling Consortium. The potential effect of improved provision of rabies post-exposure prophylaxis in Gavi-eligible countries: a modelling study. Lancet Infect Dis. 2019; 19(1):102-111.
13. Chen Q. Accelerate the Progress Towards Elimination of Dog-Mediated Rabies in China. China CDC Wkly. 2021,3(39):813-814.
14. Wang D, Zhang X, Wang X, Wang Y, Zhang R, Chen Y, et al. Cost-effectiveness analysis of rabies immunization strategy based on dynamic-decision tree model. Chi J Pre Med. 2019; 53(8):804-810. (in Chinese)
15. World Health Organization, Food and Agriculture Organization of the United Nations & World Organisation for Animal Health. Zero by 30: the global strategic plan to end human deaths from dog-mediated rabies by 2030. Geneva: WHO press; 2018.

## Table S3. The parameter values under scenarios using different strategies for rabies control in China

| **Scenarios** | **Rabid incidence in dogs** | **Probability of seeking medical care after being bitten** | **Probability of receiving PEP if treatment sought** | **Probability of completing PEP regimen if PEP received** |
| --- | --- | --- | --- | --- |
| Scenario 2 | same as *scenario 1* | with 0.01 increment per year to a cap of 0.90 | with 0.01 increment per year to a cap of 0.99 | with 0.01 increment per year to a cap of 0.975 |
| Scenario 3a | decrease with a trajectory | same as *scenario 1* | same as *scenario 1* | same as *scenario 1* |
| Scenario 3b | same as *scenario 3a* | same as *scenario 2* | same as *scenario 2* | same as *scenario 2* |
| Scenario 4a | same as *scenario 1* | same as *scenario 1* | 50% of healthy bite patients will receive the PEP vaccine if rabies is still endemic;  10% of healthy bite patients will receive the PEP vaccine if rabies is eliminated. | same as *scenario 1* |
| Scenario 4b | same as *scenario 1* | same as *scenario 2* | same as *scenario 4a* | same as *scenario 2* |
| Scenario 4c | same as *scenario 3a* | same as *scenario 1* | same as *scenario 4a* | same as *scenario 1* |
| Scenario 4d | same as *scenario 3a* | same as *scenario 2* | same as *scenario 4a* | same as *scenario 2* |

*Scenario 1*, status quo; *Scenario 2*, improved PEP; *Scenario 3a*, improved mass dog vaccination; *Scenario 3b*, improved PEP + improved mass dog vaccination; *Scenario 4a*, Status Quo + IBCM; *Scenario 4b*, improved PEP + IBCM; *Scenario 4c*, improved mass dog vaccination + IBCM; *Scenario 4d*, improved PEP + improved mass dog vaccination + IBCM. PEP, post-exposure prophylaxis; IBCM, integrated bite case management.

## Table S4. The region-specific parameters under the scenario of the status quo (*Scenario 1*)

| **Region** | **Probability of seeking medical care after being bitten** | **Probability of receiving PEP if treatment sought** | **Probability of completing PEP regimen if PEP received** | **Probability of bitten by healthy dog** | **Proportions of exposure category III** | **Probability of receiving RIG in patients with exposure category III** |
| --- | --- | --- | --- | --- | --- | --- |
| China (Overall) | 0.85 | 0.99 | 0.91 | 0.005503 | 0.425 | 0.405 |
| Shandong (East China) | 0.93 | 1.00 | 0.73 | 0.006835 | 0.537 | 0.362 |
| Hunan (Central China) | 0.76 | 0.99 | 0.98 | 0.008849 | 0.468 | 0.494 |
| Tianjin (North China) | 0.85 | 0.97 | 0.95 | 0.007500 | 0.502 | 0.256 |
| Guangxi (South China) | 0.85 | 0.99 | 0.84 | 0.012072 | 0.229 | 0.471 |
| Shaanxi (Northwest China) | 0.85 | 0.99 | 0.91 | 0.004683 | 0.693 | 0.402 |
| Guizhou (Southwest China) | 0.85 | 0.99 | 0.96 | 0.006654 | 0.557 | 0.360 |

PEP, post-exposure prophylaxis; RIG, rabies immunoglobulin.

## Table S5. The life table for the estimation of life expectancy

| **Year** | **Age (x)** | **Age interval (n)** | **Central death rate m(x,n)** | **Probability of dying q(x,n)** | **Probability of surviving p(x,n)** | **Number of survivors l(x)** | **Number of deaths d(x,n)** | **Number of person-years lived L(x,n)** | **Survival ratio S(x,n)** | **Person-years lived T(x)** | **Expectation of life e(x)** |
| --- | --- | --- | --- | --- | --- | --- | --- | --- | --- | --- | --- |
| 2024 | 0 | 1 | 0.00554 | 0.00552 | 0.99448 | 100 000 | 551.7 | 99524.6 | 0.99411 | 7 899 319 | 78.99 |
| 2024 | 1 | 4 | 0.00029 | 0.00117 | 0.99883 | 99 448 | 116.5 | 397529.1 | 0.99866 | 7 799 794 | 78.43 |
| 2024 | 5 | 5 | 0.00023 | 0.00117 | 0.99883 | 99 332 | 116.2 | 496389.6 | 0.99867 | 7 402 265 | 74.52 |
| 2024 | 10 | 5 | 0.00028 | 0.00142 | 0.99858 | 99 216 | 140.5 | 495730.1 | 0.99844 | 6 905 875 | 69.60 |
| 2024 | 15 | 5 | 0.00036 | 0.00182 | 0.99818 | 99 075 | 180.0 | 494955.5 | 0.99777 | 6 410 145 | 64.70 |
| 2024 | 20 | 5 | 0.00053 | 0.00262 | 0.99738 | 98 895 | 259.6 | 493851.9 | 0.99701 | 5 915 190 | 59.81 |
| 2024 | 25 | 5 | 0.00067 | 0.00334 | 0.99666 | 98 635 | 329.9 | 492376.0 | 0.99644 | 5 421 338 | 54.96 |
| 2024 | 30 | 5 | 0.00074 | 0.00370 | 0.99630 | 98 306 | 363.8 | 490625.1 | 0.99606 | 4 928 962 | 50.14 |
| 2024 | 35 | 5 | 0.00088 | 0.00439 | 0.99561 | 97 942 | 430.0 | 488693.3 | 0.99442 | 4 438 337 | 45.32 |
| 2024 | 40 | 5 | 0.00142 | 0.00707 | 0.99293 | 97 512 | 689.2 | 485968.8 | 0.99113 | 3 949 643 | 40.50 |
| 2024 | 45 | 5 | 0.00219 | 0.01090 | 0.98910 | 96 822 | 1055.3 | 481660.6 | 0.98563 | 3 463 675 | 35.77 |
| 2024 | 50 | 5 | 0.00370 | 0.01836 | 0.98164 | 95 767 | 1758.5 | 474740.8 | 0.97776 | 2 982 014 | 31.14 |
| 2024 | 55 | 5 | 0.00541 | 0.02673 | 0.97327 | 94 009 | 2512.9 | 464183.9 | 0.96450 | 2 507 273 | 26.67 |
| 2024 | 60 | 5 | 0.00953 | 0.04663 | 0.95337 | 91 496 | 4266.5 | 447707.2 | 0.94077 | 2 043 089 | 22.33 |
| 2024 | 65 | 5 | 0.01502 | 0.07250 | 0.92750 | 87 229 | 6324.2 | 421188.5 | 0.90596 | 1 595 382 | 18.29 |
| 2024 | 70 | 5 | 0.02551 | 0.12033 | 0.87967 | 80 905 | 9735.6 | 381579.9 | 0.84742 | 1 174 194 | 14.51 |
| 2024 | 75 | 5 | 0.04249 | 0.19306 | 0.80694 | 71 170 | 13740.0 | 323359.0 | 0.74981 | 792 614 | 11.14 |
| 2024 | 80 | 5 | 0.07620 | 0.32172 | 0.67828 | 57 430 | 18476.0 | 242457.9 | 0.60379 | 469 255 | 8.17 |
| 2024 | 85 | 5 | 0.12901 | 0.48485 | 0.51515 | 38 954 | 18886.6 | 146392.7 | 0.43132 | 226 797 | 5.82 |
| 2024 | 90 | 5 | 0.21697 | 0.68270 | 0.31730 | 20 067 | 13699.7 | 63141.5 | 0.24265 | 80 404 | 4.01 |
| 2024 | 95 | 5 | 0.35387 | 0.85153 | 0.14847 | 6 367 | 5421.9 | 15321.6 | 0.11244 | 17 263 | 2.71 |
| 2024 | 100 | -1 | 0.48707 | 1.00000 | 0 | 945 | 945.4 | 1941.0 | 0 | 1 941 | 2.05 |

The life table was obtained from United Nations World Population Prospects 2022 (<https://population.un.org/wpp/Download/Standard/Mortality/>).

## Table S6. The predicted number of dog-mediated human rabies deaths in different scenarios with IBCM during 2023-2035 in China by regions

| **Scenario** | **Scenario 4a** | **Scenario 4b** | **Scenario 4c** | **Scenario 4d** |
| --- | --- | --- | --- | --- |
| **China (Overall)** | | | | |
| Year 2023 | 305 (212-407) | 305 (212-407) | 306 (217-417) | 306 (217-417) |
| Year 2025 | 306 (213-408) | 268 (186-358) | 116 (19-249) | 102 (17-218) |
| Year 2028 | 307 (214-410) | 212 (147-284) | 10 (0-54) | 7 (0-37) |
| Year 2030 | 308 (214-411) | 212 (148-284) | 7 (0-84) | 5 (0-58) |
| Year 2033 | 310 (215-413) | 213 (148-286) | 0 (0-3) | 0 (0-2) |
| **Shandong (East China)** | | | | |
| Year 2023 | 10 (7-14) | 10 (7-14) | 10 (7-14) | 10 (7-14) |
| Year 2025 | 10 (7-14) | 10 (7-14) | 4 (1-8) | 4 (1-8) |
| Year 2028 | 10 (7-14) | 10 (7-14) | 0 (0-2) | 0 (0-2) |
| **Hunan (Central China)** | | | | |
| Year 2023 | 22 (16-30) | 22 (16-30) | 22 (15-30) | 22 (15-30) |
| Year 2025 | 22 (16-30) | 21 (14-28) | 9 (1-17) | 8 (1-16) |
| Year 2029 | 22 (16-30) | 17 (12-23) | 0 (0-5) | 0 (0-3) |
| **Tianjin (North China)** | | | | |
| Year 2023 | 3 (2-4) | 3 (2-4) | 3 (2-4) | 3 (2-4) |
| Year 2025 | 3 (2-4) | 2 (1-3) | 1 (0-2) | 1 (0-2) |
| Year 2026 | 3 (2-4) | 2 (1-3) | 0 (0-2) | 0 (0-1) |
| **Guangxi (South China)** | | | | |
| Year 2023 | 11 (8-15) | 11 (8-15) | 11 (8-15) | 11 (8-15) |
| Year 2025 | 11 (8-15) | 10 (7-13) | 4 (1-9) | 4 (1-8) |
| Year 2028 | 11 (8-15) | 8 (5-10) | 0 (0-2) | 0 (0-1) |
| **Shaanxi (Northwest China)** | | | | |
| Year 2023 | 8 (6-12) | 8 (6-12) | 9 (6-12) | 9 (6-12) |
| Year 2025 | 9 (6-12) | 7 (5-10) | 3 (0-7) | 3 (0-6) |
| Year 2028 | 9 (6-12) | 6 (4-8) | 0 (0-1) | 0 (0-1) |
| **Guizhou (Southwest China)** | | | | |
| Year 2023 | 8 (6-11) | 8 (6-11) | 8 (6-11) | 8 (6-11) |
| Year 2025 | 8 (6-11) | 7 (5-10) | 3 (0-7) | 3 (0-6) |
| Year 2027 | 8 (6-11) | 6 (4-8) | 1 (0-3) | 0 (0-2) |

Scenario 4a, Status Quo + IBCM; Scenario 4b, Improved PEP + IBCM; Scenario 4c, Improved mass dog vaccination + IBCM; Scenario 4d, Improved PEP + improved mass dog vaccination +IBCM. PEP, post-exposure prophylaxis; IBCM, integrated bite case management.

## Table S7. The incremental cost-effectiveness ratio per death prevented by different strategies compared with the status quo in China during 2024-2035

| **Scenario** | **The difference in deaths** | **The difference in costs**  **(US dollars, in billions)** | **ICER**  **(US dollars per death prevented, in millions)** |
| --- | --- | --- | --- |
| Scenario 2 | -958 | 0.3236 | 0.3378 |
| Scenario 3a | -3265 | 3.1442 | 0.9629 |
| Scenario 3b | -3318 | 3.4689 | 1.0453 |
| Scenario 4a | 0 | -2.6048 | Not Applicable |
| Scenario 4b | -958 | -2.4428 | dominate |
| Scenario 4c | -3265 | -0.6497 | dominate |
| Scenario 4d | -3318 | -0.5775 | dominate |

China's per capita gross domestic product in 2020 is 10410.4586 US dollars. All scenarios are compared to the status quo. *Scenario 2*, improved PEP; *Scenario 3a*, improved mass dog vaccination; *Scenario 3b*, improved PEP + improved mass dog vaccination; *Scenario 4a*, Status Quo + IBCM; *Scenario 4b*, improved PEP + IBCM; *Scenario 4c*, improved mass dog vaccination + IBCM; *Scenario 4d*, improved PEP + improved mass dog vaccination + IBCM. PEP, post-exposure prophylaxis; IBCM, integrated bite case management; ICER, incremental cost-effectiveness ratio.

## Table S8. The ICER per death prevented by different strategies compared with the status quo during 2024-2035 in Shandong (East China)

| **Scenario** | **The difference in deaths** | **The difference in costs**  **(US dollars, in billions)** | **ICER**  **(US dollars per death prevented, in millions)** |
| --- | --- | --- | --- |
| Scenario 2 | -1 | 0.0093 | 7.5620 |
| Scenario 3a | -109 | 0.2242 | 2.0634 |
| Scenario 3b | -109 | 0.2335 | 2.1481 |
| Scenario 4a | 0 | -0.2307 | Not Applicable |
| Scenario 4b | -1 | -0.2261 | dominate |
| Scenario 4c | -109 | -0.1100 | dominate |
| Scenario 4d | -109 | -0.1082 | dominate |

Shandong's per capita gross domestic product in 2020 is 10457.2729 US dollars. All scenarios are compared to the status quo. *Scenario 2*, improved PEP; *Scenario 3a*, improved mass dog vaccination; *Scenario 3b*, improved PEP + improved mass dog vaccination; *Scenario 4a*, Status Quo + IBCM; *Scenario 4b*, improved PEP + IBCM; *Scenario 4c*, improved mass dog vaccination + IBCM; *Scenario 4d*, improved PEP + improved mass dog vaccination + IBCM. PEP, post-exposure prophylaxis; IBCM, integrated bite case management; ICER, incremental cost-effectiveness ratio.

## Table S9. The ICER per death prevented by different strategies compared with the status quo during 2024-2035 in Hunan (Central China)

| **Scenario** | **The difference in deaths** | **The difference in costs**  **(US dollars, in billions)** | **ICER**  **(US dollars per death prevented, in millions)** |
| --- | --- | --- | --- |
| Scenario 2 | -70 | 0.0333 | 0.4780 |
| Scenario 3a | -238 | 0.1468 | 0.6161 |
| Scenario 3b | -241 | 0.1801 | 0.7478 |
| Scenario 4a | 0 | -0.2054 | Not Applicable |
| Scenario 4b | -70 | -0.1887 | dominate |
| Scenario 4c | -238 | -0.1521 | dominate |
| Scenario 4d | -241 | -0.1459 | dominate |

Hunan's per capita gross domestic product in 2020 is 9116.4705 US dollars. All scenarios are compared to the status quo. *Scenario 2*, improved PEP; *Scenario 3a*, improved mass dog vaccination; *Scenario 3b*, improved PEP + improved mass dog vaccination; *Scenario 4a*, Status Quo + IBCM; *Scenario 4b*, improved PEP + IBCM; *Scenario 4c*, improved mass dog vaccination + IBCM; *Scenario 4d*, improved PEP + improved mass dog vaccination + IBCM. PEP, post-exposure prophylaxis; IBCM, integrated bite case management; ICER, incremental cost-effectiveness ratio.

## Table S10. The ICER per death prevented by different strategies compared with the status quo during 2024-2035 in Tianjin (North China)

| **Scenario** | **The difference in deaths** | **The difference in costs**  **(US dollars, in billions)** | **ICER**  **(US dollars per death prevented, in millions)** |
| --- | --- | --- | --- |
| Scenario 2 | -10 | 0.0046 | 0.442 |
| Scenario 3a | -28 | 0.0244 | 0.8595 |
| Scenario 3b | -29 | 0.029 | 0.9979 |
| Scenario 4a | 0 | -0.0308 | Not Applicable |
| Scenario 4b | -10 | -0.0285 | dominate |
| Scenario 4c | -28 | -0.0204 | dominate |
| Scenario 4d | -29 | -0.0193 | dominate |

Tianjin's per capita gross domestic product in 2020 is 14727.5204 US dollars. All scenarios are compared to the status quo. *Scenario 2*, improved PEP; *Scenario 3a*, improved mass dog vaccination; *Scenario 3b*, improved PEP + improved mass dog vaccination; *Scenario 4a*, Status Quo + IBCM; *Scenario 4b*, improved PEP + IBCM; *Scenario 4c*, improved mass dog vaccination + IBCM; *Scenario 4d*, improved PEP + improved mass dog vaccination + IBCM. PEP, post-exposure prophylaxis; IBCM, integrated bite case management; ICER, incremental cost-effectiveness ratio.

## Table S11. The ICER per death prevented by different strategies compared with the status quo during 2024-2035 in Guangxi (South China)

| **Scenario** | **The difference in deaths** | **The difference in costs**  **(US dollars, in billions)** | **ICER**  **(US dollars per death prevented, in millions)** |
| --- | --- | --- | --- |
| Scenario 2 | -34 | 0.0246 | 0.7238 |
| Scenario 3a | -116 | 0.1121 | 0.9650 |
| Scenario 3b | -118 | 0.1367 | 1.1593 |
| Scenario 4a | 0 | -0.1818 | Not Applicable |
| Scenario 4b | -34 | -0.1695 | dominate |
| Scenario 4c | -116 | -0.1542 | dominate |
| Scenario 4d | -118 | -0.1491 | dominate |

Guangxi's per capita gross domestic product in 2020 is 6421.9665 US dollars. All scenarios are compared to the status quo. *Scenario 2*, improved PEP; *Scenario 3a*, improved mass dog vaccination; *Scenario 3b*, improved PEP + improved mass dog vaccination; *Scenario 4a*, Status Quo + IBCM; *Scenario 4b*, improved PEP + IBCM; *Scenario 4c*, improved mass dog vaccination + IBCM; *Scenario 4d*, improved PEP + improved mass dog vaccination + IBCM. PEP, post-exposure prophylaxis; IBCM, integrated bite case management; ICER, incremental cost-effectiveness ratio.

## Table S12. The ICER per death prevented by different strategies compared with the status quo during 2024-2035 in Shaanxi (Northwest China)

| **Scenario** | **The difference in deaths** | **The difference in costs**  **(US dollars, in billions)** | **ICER**  **(US dollars per death prevented, in millions)** |
| --- | --- | --- | --- |
| Scenario 2 | -27 | 0.0093 | 0.348 |
| Scenario 3a | -91 | 0.087 | 0.9568 |
| Scenario 3b | -92 | 0.0963 | 1.0421 |
| Scenario 4a | 0 | -0.0795 | Not Applicable |
| Scenario 4b | -27 | -0.0748 | dominate |
| Scenario 4c | -91 | -0.0284 | dominate |
| Scenario 4d | -92 | -0.0263 | dominate |

Shaanxi's per capita gross domestic product in 2020 is 9608.0932 US dollars. All scenarios are compared to the status quo. *Scenario 2*, improved PEP; *Scenario 3a*, improved mass dog vaccination; *Scenario 3b*, improved PEP + improved mass dog vaccination; *Scenario 4a*, Status Quo + IBCM; *Scenario 4b*, improved PEP + IBCM; *Scenario 4c*, improved mass dog vaccination + IBCM; *Scenario 4d*, improved PEP + improved mass dog vaccination + IBCM. PEP, post-exposure prophylaxis; IBCM, integrated bite case management; ICER, incremental cost-effectiveness ratio.

## Table S13. The ICER per death prevented by different strategies compared with the status quo during 2024-2035 in Guizhou (Southwest China)

| **Scenario** | **The difference in deaths** | **The difference in costs**  **(US dollars, in billions)** | **ICER**  **(US dollars per death prevented, in millions)** |
| --- | --- | --- | --- |
| Scenario 2 | -26 | 0.0103 | 0.3930 |
| Scenario 3a | -90 | 0.0851 | 0.9496 |
| Scenario 3b | -91 | 0.0953 | 1.0475 |
| Scenario 4a | 0 | -0.0972 | Not Applicable |
| Scenario 4b | -26 | -0.0921 | dominate |
| Scenario 4c | -90 | -0.0563 | dominate |
| Scenario 4d | -91 | -0.0540 | dominate |

Guizhou's per capita gross domestic product in 2020 is 6705.7511 US dollars. All scenarios are compared to the status quo. *Scenario 2*, improved PEP; *Scenario 3a*, improved mass dog vaccination; *Scenario 3b*, improved PEP + improved mass dog vaccination; *Scenario 4a*, Status Quo + IBCM; *Scenario 4b*, improved PEP + IBCM; *Scenario 4c*, improved mass dog vaccination + IBCM; *Scenario 4d*, improved PEP + improved mass dog vaccination + IBCM. PEP, post-exposure prophylaxis; IBCM, integrated bite case management; ICER, incremental cost-effectiveness ratio.
